# Ecosystem functioning in urban grasslands: The role of biodiversity, plant invasions and urbanization

**DOI:** 10.1371/journal.pone.0225438

**Published:** 2019-11-22

**Authors:** Gabriela Onandia, Conrad Schittko, Masahiro Ryo, Maud Bernard-Verdier, Tina Heger, Jasmin Joshi, Ingo Kowarik, Arthur Gessler

**Affiliations:** 1 Research Platform “Data”, Leibniz Centre for Agricultural Landscape Research (ZALF), Müncheberg, Germany; 2 Berlin-Brandenburg Institute of Advanced Biodiversity Research (BBIB), Berlin, Germany; 3 Biodiversity Research and Systematic Botany, University of Potsdam, Potsdam, Germany; 4 Institute of Biology, Freie Universität Berlin, Berlin, Germany; 5 Division of Zoology, Freie Universität Berlin, Berlin, Germany; 6 Restoration Ecology, Technical University of Munich, Freising, Germany; 7 Institute for Landscape and Open Space, HSR Hochschule für Technik, Rapperswil, Switzerland; 8 Department of Ecology, Ecosystem Science and Plant Ecology, Technische Universität Berlin, Berlin, Germany; 9 Department of Forest Dynamics, Swiss Federal Research Institute for Forest, Snow and Landscape Research WSL, Birmensdorf, Switzerland; 10 Department of Environmental Systems Science, ETH Zurich, Zurich, Switzerland; The University of Auckland, NEW ZEALAND

## Abstract

Urbanization is driving the transformation of natural and rural ecosystems worldwide by affecting both the abiotic environment and the biota. This raises the question whether urban ecosystems are able to provide services in a comparable way to their non-urban counterparts. In urban grasslands, the effects of urbanization-driven ecological novelty and the role of plant diversity in modulating ecosystem functioning have received little attention. In this study, we assessed the influence of biodiversity, abiotic and biotic novelty on ecosystem functioning based on *in situ* measurements in non-manipulated grasslands along an urbanization gradient in Berlin (Germany). We focused on plant aboveground biomass (AGB), intrinsic water-use efficiency (iWUE) and ^15^N enrichment factor (Δδ^15^N) as proxies for biomass production, water and N cycling, respectively, within grassland communities, and tested how they change with plant biogeographic status (native *vs* alien), functional group and species identity. Approximately one third of the forb species were alien to Berlin and they were responsible for 13.1% of community AGB. Community AGB was positively correlated with plant-species richness. In contrast, iWUE and Δδ^15^N were mostly determined by light availability (depicted by sky view factor) and urban parameters like the percentage of impervious surface or human population density. We found that abiotic novelty potentially favors aliens in Berlin, mainly by enhancing their dispersal and fitness under drought. Mainly urban parameters indicating abiotic novelty were significantly correlated to both alien and native Δδ^15^N, but to AGB and iWUE of alien plants only, pointing to a stronger impact of abiotic novelty on N cycling compared to C and water cycling. At the species level, sky view factor appeared to be the prevailing driver of photosynthetic performance and resource-use efficiency. Although we identified a significant impact of abiotic novelty on AGB, iWUE and Δδ^15^N at different levels, the relationship between species richness and community AGB found in the urban grasslands studied in Berlin was comparable to that described in non-urban experimental grasslands in Europe. Hence, our results indicate that conserving and enhancing biodiversity in urban ecosystems is essential to preserve ecosystem services related to AGB production. For ensuring the provision of ecosystem services associated to water and N use, however, changes in urban abiotic parameters seem necessary.

## Introduction

Increasing human population and urban sprawl are driving the fast transformation of natural and rural environments into urban ecosystems worldwide [1]. Over the past six decades, 25% of the global population has moved from rural into urban settlements and this trend is expected to continue [2]. Urbanization encompasses significant changes in all components of urban ecosystems and their functioning [3], such as the so-called urban heat island effect [4], higher concentrations of heavy metals in the soil and soil compaction [5–7] increased atmospheric nitrogen deposition [8], habitat fragmentation and isolation [9] as well as the development of transportation networks that favor the dispersal of alien plants [10, 11]. Urbanization is also a powerful driver of change to biodiversity patterns, associated with filtering species according to their pre-adaptation to urban environments [12–14]. While alien plant species are frequently found in different urban settings, particularly in novel urban ecosystems [15], cities can also harbor a considerable richness of native plant species [12, 16]. However, urbanization has been also linked to a loss of biodiversity, ecological homogenization and changes in community composition, including a higher proportion of alien species pre-adapted to novel habitats [17–20] and species compositions that cannot be found in near-natural environments.

Consequently, species in urbanized settings face situations to which they have not been exposed during their evolutionary history. Urbanization leads to the transformation of (near-)natural into novel urban ecosystems that prevail in many cities and can be characterized by both novel habitat configurations and novel species assemblages without analogues to natural landscapes [18]. These conditions thus can be characterized as ‘ecologically novel’ [21], and ‘ecological novelty’ in this sense can be seen as an overarching conceptual framework to address the complex eco-evolutionary consequences of anthropogenically induced changes for organisms and ecosystems. In the following, we use the term ‘abiotic novelty’ to characterize environmental conditions that differ from near-natural grassland conditions typical for a particular area. ‘Biotic novelty’ refers to the presence of species, and thus interaction partners, that have not been present in the area in historic times (i.e. alien species).

Grasslands in rural settings provide a wide variety of relevant ecosystem services such as microclimate regulation, water flow regulation and nutrient cycling [22]. Grassland vegetation supports the sequestration of carbon dioxide and soil formation through primary production and modulates hydrological flows by fluctuations in its water use. Moreover, through the acquisition and storage of nitrogen (N), grassland vegetation plays a major role in terrestrial nitrogen cycling. Worldwide, grasslands make up a considerable area in cities. In Berlin, for instance, they cover as much as 5% of the city area [23]. Urban grasslands encompass meadows and lawns in domestic gardens, parks, vacant land, remnants of rural landscapes, and areas along transportation corridors [23–28]. The role of urban grasslands for biodiversity conservation has gained increasing attention in the last decade [23, 24, 29, 30]. Yet in urban grasslands, the role of plant diversity in modulating ecosystem functioning, including proxies such as aboveground biomass (AGB) production or resource use has received little attention [24, 31].

A positive relationship between biodiversity and ecosystem functioning (often exemplified by AGB and less frequently by plant water or N use) has been widely described in non-urban experimental grasslands resembling natural or agricultural grassland communities [22, 32–39]. This relationship has been also found in experimental urban grassland assemblages resembling North America turfgrass communities [40] and experimental grassland assemblages resembling the soil and vegetation on abandoned urban sites in Berlin [41]. However, it remains unknown whether the positive link between biodiversity and ecosystem functioning prevails in urban grasslands where biodiversity and other environmental factors are not manipulated (i.e. non-experimental grasslands)[42]. Ecosystem functioning in urban grasslands is critically understudied, with only a few studies focusing on urban lawns as a subgroup of urban grasslands (e.g. [20, 40, 43, 44]). Furthermore, studies comparing ecosystem functions of urban *vs* non-urban grasslands are rare (but see [43, 44]). One of these few studies has shown that in the Front Range of Colorado (USA), lawns were more productive than native grasslands, likely as a result of increased irrigation or fertilization associated to management [44].

Along with biodiversity, both biotic novelty (the presence of alien species) and abiotic novelty (altered environmental conditions) can be expected to ultimately affect ecosystem services by modifying ecosystem functioning. For example, strongly altered urban soils may be less productive than rural soils; and a high abundance of alien plants may modulate biomass production or nutrient cycling–with negative effects on ecosystem services [45]. Plant invasions have been often associated with negative effects on native plant and animal species, ecological functions and ecosystem services [45–47]. For instance, a global meta-analysis of field studies assessing the impact of alien plants revealed that overall species diversity declined but primary production increased, probably due to a sampling effect [47]. Yet, invasion effects on ecological functions are clearly understudied in urban environments (e.g., [31, 46]). Given the high abundance of alien plants in urban environments, and particularly in novel urban ecosystems [15], enhanced insights into biodiversity-ecosystem functioning relationships in cities are important for urban planning and biodiversity conservation, and ultimately for human well-being.

Nonetheless, the available information on the role of ecological novelty in influencing ecosystem functioning proxies such as AGB production, intrinsic water-use efficiency (iWUE) and N cycling in urban grasslands is limited. Furthermore, the relative role of biotic versus abiotic factors, and their significance in comparison to biodiversity as a driver of ecosystem functioning is unclear. Evidence on the key drivers of various ecosystem properties compiled for a wide range of aquatic and terrestrial ecosystems is diverse ([36] and references therein): biotic changes were generally found to have an equally important effect on ecosystem properties than abiotic changes [48]. For instance, in boreal forests, changes in microclimate (i.e. decreased heat flux into the soil) induced by moss cover may modulate permafrost stability in a comparable way to environmental change. Conversely, abiotic conditions may sometimes offset the effects of species richness on ecosystem processes, as shown for instance by a theoretical modelling study revealing that abiotic conditions mask the effect of plant species richness on plant productivity [49]. Such ambiguity may be due to different mechanisms acting upon different groups of plant species, such as functional groups or native versus alien species, or different mechanisms acting during different periods of the growing season.

Despite the fact that the performance of individual species, or functional groups of species, might ultimately determine ecosystem level functioning (e.g. [50, 51]), current knowledge on species-specific responses to biodiversity and ecological novelty [52] and on seasonal variations in such response is scarce, especially in the context of urban grassland research. In this regard, the combined estimation of multiple photosynthetic parameters as well as leaf stable isotope composition can provide insights into the physiological mechanisms controlling plant individual´s performance and AGB production, especially when measured over the course of the growing season [53]. Chlorophyll fluorescence, for instance, reflects the plant ability to put up with abiotic stress factors and indicates the damage level of the photosynthetic apparatus [54], while leaf C isotope composition reflects the average intrinsic water use efficiency throughout the time period during which the organic matter assessed was formed [55].

Based on the positive relationship between diversity and ecosystem functioning found in numerous experimental grasslands and semi-natural grasslands, namely between diversity and AGB production [22, 33, 37, 40], diversity and water use [50, 56] as well as diversity and N cycling [57], we hypothesize that species richness is the main controlling factor for plant community AGB, iWUE and N use in urban grasslands, overriding both abiotic and biotic novelty effects. In this study we aim to improve the understanding of the relative importance of biodiversity, abiotic and biotic novelty for the functioning of urban grasslands. To our knowledge, this is the first study to do so based on *in situ* measurements in non-manipulated urban grasslands. Indeed, previous results based on experimental urban grasslands pointed to the importance of such field assessments [40]. In particular, we aim to:

- identify the main factors (i.e. parameters related to biodiversity, abiotic and biotic novelty) driving the variability in AGB, iWUE and N cycling of whole plant communities, plant functional groups and plants with different biogeographic status (native *vs* alien) across 20 urban grasslands located in Berlin;
- assess the biodiversity-ecosystem functioning relationship in the studied urban grasslands and compare it with that found in natural and agricultural grasslands;
- test if parameters related to AGB production, iWUE and N use at the species level significantly differ between the mid- and peak growing season and assess the relationship between those parameters and biodiversity, abiotic and biotic novelty in both time periods.

## Material and methods

### Study area and study system

**Fig 1:**
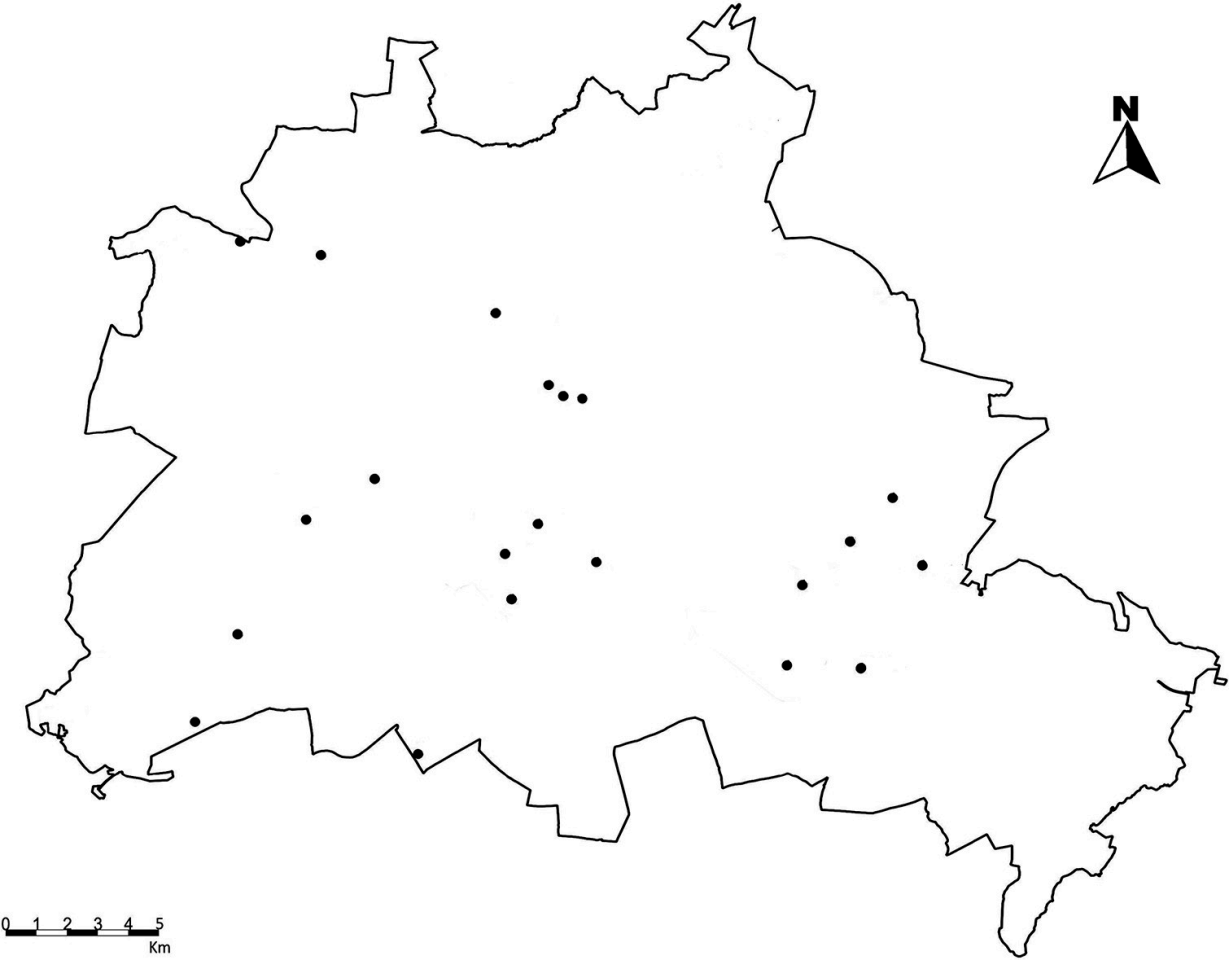
Geographical location of the 20 grasslands studied in Berlin (Germany) in 2017.

The study was carried out in Berlin, Germany. Berlin has an area of 891.1 km^2^ and a population of ≈ 3.6 mio. inhabitants [58]. Mean annual precipitation amounted to 576 mm and mean annual air temperature was 9.9°C for the period from 1981 to 2010 at the central city location of Tempelhof [59]. Our study system is dry grasslands according to the classification of the “Catalogue of Natural Habitats and Species of Appendices I and II of the Habitats Directive in Brandenburg” [60]. Dry grasslands are a vegetation type that spans a range of near-natural to strongly human-influenced sites throughout the city. For this reason, urban dry grasslands had been selected as a model ecosystem within the *CityScapeLabs*, an experimental platform established by the Berlin-Brandenburg Institute of Advanced Biodiversity Research (BBIB) with a network of permanent plots for the investigation of biodiversity and ecosystem functioning in urban environments [61]. For this study, we selected a subset of 20 dry grassland plots of 16 m^2^ that were relatively evenly distributed across the city (Fig 1) and whose surroundings were subject to different levels of urbanization, indicated, e.g. by human population density or percentage of impervious (sealed) surface. These plots comprised several land use types (e.g. parks, cemeteries, forest clearings), but were selected to minimize human management inputs such as fertilization, irrigation and mowing. The parameters related to biodiversity and abiotic novelty at the study sites (with the exception of air temperature and air relative humidity) were provided by the *CityScapeLabs*, and the degree of biotic novelty of the study sites was calculated based on the *CityScapeLab*s biodiversity data (see Table 1 for more information).

**Table 1:** Parameters related to biodiversity, abiotic novelty and biotic novelty in the studied grasslands in Berlin.

Parameter (with categories or range if applicable)	Description	Min-Max	Average ± SD
**Abiotic novelty**			
**Habitat continuity and connectivity**			
Age: N = new (since 1945); O = old (since 1831–1940)	Historical continuity as dry grassland biotope	O, N	-
Size of dry grassland biotope patch (m^2^)	Size of dry grassland biotope patch in which the plot is located	335–19058	6603 ± 5770
Share of grassland_100/_500/_1000/_5000 (0–1)	Proportion of dry grassland biotope in a 100/500/1000/5000 m buffer radii around the dry grassland biotope patch in which the plot is located		
Share of grassland_100		0–0.28	0.076 ± 0.096
Share of grassland_500		0–0.15	0.027 ± 0.037
Share of grassland_1000		0–0.11	0.015 ± 0.025
Share of grassland_5000		0–0.02	0.007 ± 0.007
**Urban parameters**			
Impervious surface_100/_500/_1000/_5000 (%)	Percentage of sealed surface in a 100/500/1000/5000 m buffer radii around the dry grassland biotope patch in which the plot is located		
Impervious surface_100		0–70.3	23.7 ± 22.6
Impervious surface_500		1.2–67.6	31.6 ± 23.4
Impervious surface_1000		1.1–71.2	36 ± 22.6
Impervious surface_5000		14.1–63.1	40.5 ± 16.4
FAR_100/_500/_1000/_5000	Ratio of a building´s total floor area to the land area upon which it is built in a 100, 500, 1000 and 5000 m buffer radii around the dry grassland biotope patch in which the plot is located		
FAR_100		0–1.21	0.17 ± 0.29
FAR_500		0–1.1	0.31 ± 0.34
FAR_1000		0–1.28	0.4 ± 0.4
FAR_5000		0.1–1.1	0.49 ± 0.35
PopD_100/_500/_1000/_5000 (inhabitants ha^-1^)	Population density in a 100/500/1000/5000 m buffer radii around the dry grassland biotope patch in which the plot is located		
PopD_100		0–97.1	15.3 ± 30.4
PopD_500		0–105.3	33.4 ± 37.8
PopD_1000		0.1–125.8	43.2 ± 38.9
PopD_5000		10.2–106.3	51.8 ± 33.6
RoadD_100/_500/_1000/_5000 (km)	Road density: total length of roads in a 100/500/1000/5000 m buffer radii around the dry grassland biotope patch in which the plot is located.		
RoadD_100		0–2.2	0.5 ± 0.7
RoadD_500		0.2–24.7	8.5 ± 6.9
RoadD_1000		2.7–67.6	33.9 ± 18.4
RoadD_5000		385.7–1226.4	860.6 ± 263.7
RailwD_100/_500/_1000/_5000 (km)	Railway density: total length of railways in a 100/500/1000/5000 m buffer radii around the dry grassland biotope patch in which the plot is located		
RailwD_100		0–2.6	0.2 ± 0.6
RailwD_500		0–8.4	1.5 ± 2.5
RailwD_1000		0–29	5 ± 7
RailwD_5000		11.5–210.5	99.3 ± 53.3
RDist (m)	Shortest distance from plot midpoint to nearest road	15–496	144 ± 128
RailwDist (m)	Shortest distance from plot midpoint to nearest railway	15–4111	967 ± 995
**Climate and weather**			
Long-term air T (°C): >8–8.5; >8.5–9; >9–9.5; >9.5–10; >10–10.5	Air temperature zone (long- term average 1961–1990)	8–10.5	-
UrbClim: 0 = no changes; 1 = very low changes; 2 = low changes; 3 = medium changes; 4 = high changes	Urban climatic zone: changes in temperature, air moisture and wind regime compared to open land conditions	1–4	2.2 ± 1.2
AirT (°C)	Air temperature	14.3–16.7	15.4 ± 0.5
AirRH (%)	Air relative humidity	67–88	78.5 ± 4.8
SVF (0–1)	Sky view factor: share of open sky	0.68–0.99	0.88 ± 0.09
**Soil**	** **		
N (g kg^-1^)	Nitrogen content	0.01–0.60	0.1 ± 0.13
Cu (mg kg^-1^)	Copper content	0.05–0.42	0.14 ± 0.09
Zn (mg kg^-1^)	Zinc content	0.03–10.71	1.21. ± 2.58
Cd (mg kg^-1^)	Cadmium content	0.00–0.08	0.01 ± 0.02
Pb (mg kg^-1^)	Lead content	0.01–0.28	0.05 ± 0.07
Ni (mg kg^-1^)	Nickel content	0.00–0.15	0.02 ± 0.03
W_C_ (%)	Gravimetric water content (referred to dry matter)	0.01–0.29	0.06 ± 0.06
**Vegetation**	** **	** **	** **
Species richness	Number of plant species in a 16m^2^ grassland plot	13–48	27.8 ± 7.6
Rao´s Q	Functional diversity	0.05–0.11	0.07 ± 0.02
Moss cover (%)	Percentage of plot surface covered by moss	0–60	24 ± 21
Litter cover (%)	Percentage of plot surface covered by litter	10–80	42 ± 20
BNI	Biotic novelty index	0.0018–0.0924	0.0209 ± 0.0241

### Biodiversity

Vascular plant diversity was characterized both by taxonomic and functional diversity, estimated as species richness and Rao’s quadratic entropy (Rao´s Q, [62]), respectively. To assess species richness, vegetation surveys were carried out in a 4 x 4 m plot delimited within each of the 20 grasslands between April 18^th^ and May 19^th^ 2017. Based on expert knowledge and region-specific literature [63–65], plant species were classified according to their biogeographic status into native or alien. Species introduced after 1492 (neophytes) were considered ‘alien’, while species introduced before 1492 (archeophytes) and native species were merged into the category ‘native’. Thereafter, the proportion of alien species at each grassland plot was estimated. Rao's quadratic entropy was calculated using Gower distances between species pairs based on twelve plant functional traits: plant height, specific leaf area, life form, flower color, flower class, clonal growth organs, length of dispersal unit, seed mass, leaf area, leaf nitrogen content, nitrogen fixation and mycorrhizal infection [66]. Trait data were extracted from the TRY database [67] and the BiolFlor database [68]. Additionally, the percentage of plot surface covered by moss (moss cover) and litter (litter cover) was visually estimated.

### Abiotic novelty

A total number of 22 environmental variables related to abiotic novelty were assessed in the 20 selected dry grasslands plots within the *CityScapeLabs* (Table 1). Abiotic novelty-related parameters included urban parameters (i.e. human population density, impervious surface, floor area ratio, etc.), climate and soil parameters assumed to be linked to urbanization (i.e. heavy metals in the soil, long-term average air temperature, etc.) as well as habitat continuity (age) and size of dry grassland biotope patch.

The age of the grasslands was assessed by digitizing and georeferencing historical land use maps (i.e. Preußische Uraufnahme (1831–71) and Preußische Neuaufnahme (1927–40)) and intersecting them with the current biotope mapping of Berlin and Brandenburg. Data on size of dry grassland biotope patch, share of grassland, impervious surface, FAR, PopD, RoadD, RailwD, RDist, RailwDist, long-term air T and UrbClim (see Table 1 for parameter description) were extracted from the Geoportal Berlin of the Senate Department for Urban Development and Housing [69]. A mean value for buffer areas of multiple sizes around the biotope patch was calculated for share of grassland, impervious surface, FAR, PopD, RoadD and RailwD using QGIS 2.18.0 [70]. RDist and RailwDist were also calculated using QGIS 2.18.0 as the shortest distance from the grassland plot midpoint to the nearest road or railway, respectively.

To analyze soil parameters (Table 1), 15 subsamples of 30 cm depth were taken with a 1.4 cm stainless steel soil corer in the aforementioned delimited 4 x 4 m plot within each grassland in June 2017, air-dried, sieved (2 mm) and then mixed and homogenized into a single sample. Total soil N (TN) was analyzed by adsorption chromatography [71], whereas soil Cu, Zn, Cd, Pb and Ni were analyzed by inductively coupled plasma optical emission spectrometry (iCAP 6000 Spectrometer, Thermo Fisher Scientific, Dreieich, Germany) following soil extraction using ammonium nitrate solution [72]. Gravimetric soil water content (W_C_) was determined as % of the soil dry weight by oven drying the samples at 105°C until constant weight.

At each grassland plot, air temperature (AirT) and relative humidity (AirRH) were recorded every 30 minutes at a 20 cm height throughout the growing season (between April 21^th^ and 26^th^ November 26^th^, 2017) with a data logger (OMEGA, OM-EL-USB-2). Subsequently, the mean AirT and AirRH for the whole growing season was calculated. Due to logger misappropriation, it was not possible to record AirT and AirRH either partly or entirely in 6 plots, where 50–100% of the data were missing. Therefore, we collected missing data from meteorological stations located within 0.36 to 5 km to the plots [73]. At the plots where only part of the data was missing, we confirmed that AirT and AirRH from Weather Underground differed less than 5% and 12%, respectively, from the data recorded with microclimate logger data. Sky view factor (SVF) is a parameter related to the urban heat island phenomenon, with higher values indicating lower long-wave radiation emissions of built surfaces to the sky during the night [74]. SVF was estimated as the share of open sky based on the analyses of images taken using a Canon EOS 700D camera coupled to a circular fisheye (4.5 mm F2.8 EX DC HSM, Sigma) at ≈ 1.5 m height on the plots. Namely, SVF was calculated using the subversion SOLWEIG1D of the SOLWEIG model (version 2015a) [75, 76].

### Biotic novelty

The degree of biotic novelty of individual plots was estimated by a biotic novelty index (BNI). The BNI is a compound measure based on a modified version of Rao´s quadratic entropy and designed to quantify the functional ecological novelty of communities [66]. The index captures the functional diversity contributed by novel species recently arrived in the community, weighted by their relative abundance. It is based on two components: the pairwise functional distance between species, see [62, 77]) and a temporal coexistence component that weighs the functional differences between pairs of species based on how long both species have been present in the region. For example, if a given species pair consists of one native and one recently arrived alien species, the trait distance between both will receive a higher weight than the distance between a pair consisting of one native and one earlier arrived alien species. This idea is based on the finding that alien species will gradually become familiar with their interaction partner(s) over time [78–81] which may lead to a decrease of novelty in the community. The temporal coexistence component of the index was calculated from species’ residence times in the Berlin area. For the calculation of the functional diversity component after Rao, we used the same method and traits as described above. More information can be found in the S1 Appendix.

### Plant community aboveground biomass and soil collection for isotope analyses

Total standing aboveground biomass (AGB) of the plant community was sampled between July 24^th^ and August 9^th^ 2017 in the 20 selected dry grasslands in Berlin. As grasslands are regularly managed by the local authorities, protective bands were placed around the 4 x 4 m plot in early spring 2017 to prevent mowing prior to sampling. Since the plots had been last mowed in autumn 2016, the AGB collected in late summer 2017 biomass integrated the biomass production throughout the 2017 growing season, including both alive and dead plant parts. Replicate samples (n = 3) were collected by clipping the vegetation to the ground in 20 x 50 cm quadrats randomly selected within each plot [82]. After identification to the genus or species level, the collected plants were classified into three functional groups: legumes (N-fixing dicots), forbs (non-N-fixing dicots) and graminoids (grasses, sedges and rushes). Next, they were separated into biogeographic status classes (native *vs* alien). Samples were transported to the lab within 6 hours, oven dried at 70°C to constant weight and weighed thereafter (Kern EW 620, Kern & Sohn GmbH, Balingen, Germany). For each plot, the AGB of different plant groups (i.e. graminoids (AGB_G_), forbs (AGB_F_) and legumes (AGB_L_), natives (AGB_N_) aliens (AGB_N_), native graminoids (AGB_NG_), alien graminoids (AGB_AG_), native forbs (AGB_NF_), alien forbs (AGB_AF_), native legumes (AGB_NL_), alien legumes (AGB_AL_)) as well as the whole community AGB (AGB_C_) was calculated as the average of the three replicates within the plot. Following aboveground biomass collection, a soil core of 30 cm depth was extracted with a 5 cm diameter soil core sampler (Wurzelbohrer V2A, Umwelt-Geräte-Technik GmbH, Müncheberg, Germany) at every quadrat replicate (n = 3) within each grassland plot. The upper 10 cm of the soil cores were homogenized, sieved (2 mm mesh size) and oven-dried at 105°C prior to isotope analyses.

### Species level gas-exchange and chlorophyll-a fluorescence measurements

In order to assess the effects of biodiversity, abiotic novelty and biotic novelty on AGB production at the species level, we selected two plant species that were relatively common in the study area and belonged to the two dominant functional groups in terms of biomass: the grass *Calamagrostis epigejos* (L.) Roth and the forb *Plantago lanceolata* L.. Ecophysiological parameters were measured during two time periods: in spring (mid-growing season), between May 8^th^ and May 19^th^ 2017, and in summer (peak of the growing season), between July 27^th^ and August 9^th^ 2017, simultaneously to the collection of AGB and soil. Whereas in spring we selected individuals that were exclusively present in the 4 x 4 m plot delimited within each grassland, in summer we selected individuals that were also outside the plot in order to obtain a minimum number of replicates. These individuals were located within a distance of 10 m from the plot, in comparable vegetation and soil conditions and thus we do not expect them to be experiencing different conditions from the ones inside the plot. Therefore, the number of plots in which the species were present varied between months, with *C*. *epigejos* being present in 13 and 17, respectively, out of the 20 selected grassland plots in spring and summer, respectively, and *P*. *lanceolata* occurring in 9 and 12 plots, in spring and summer, respectively. In total, *C*. *epigejos* and *P*. *lanceolta* co-occurred in 40–55% of the study plots, which covered a comparable gradient of plant species richness, abiotic, and biotic novelty (S1 Table).

The ecophysiological parameters were measured with a portable gas exchange fluorescence system (Walz GFS-3000, Heinz Walz GmbH, Effeltrich, Germany) equipped with a clamp-on leaf chamber of 8 cm^2^. Gas exchange measurements were carried out mostly on clear sunny days between 09:00 and 16:30 on unshaded, fully expanded mature and healthy leaves from three individuals per species in each plot/grassland. The leaf chamber conditions were set constant to 20°C leaf temperature, 60% relative humidity, 400 ppm CO_2_ concentration and saturating photosynthetic photon flux density (PPFD_sat_, 1500 μmol m^-2^ s^-1^). Gas exchange parameters were logged after the leaves reached steady-state conditions at saturating PPFD and comprised photosynthetic rate (A, μmol CO_2_ m^-2^ s^-1^), transpiration rate (E, mmol H_2_O m^-2^ s^-1^), stomatal conductance (g_s_, mol air m^-2^ s^-1^) and ratio of the intercellular to ambient CO_2_ concentration (c_i_/c_a_, μmol CO_2_ μmol^-1^ CO_2_). Instantaneous water-use efficiency (instant-WUE, μmol CO_2_ μmol^-1^ H_2_O) was calculated as A/E. Chlorophyll-*a* fluorescence parameters were measured with a fluorescence Module (LED-Array/PAM-Fluorometer 3055-FL, Heinz Walz GmbH, Effeltrich, Germany) integrated in the leaf chamber. These parameters included steady-state fluorescence of the light-adapted leave (F), steady-state fluorescence of the dark-adapted leave (F_0_), fluorescence of the dark-adapted leaf during a saturating light pulse (F_m_), fluorescence of the illuminated leaf when a saturating light pulse is superimposed on the prevailing environmental light levels (F_m_´, [83]). The maximum potential quantum yield of photosystem II was calculated as F_v_/F_m_, where F_v_ = F_m_—F_0._ The effective quantum yield of electron transport through photosystem II was calculated as ΔF/F_m_′ = (F_m_´ - F)/F_m_´ [84]. Photosynthetic light-response curves of ΔF/F_m_′ and apparent electron transport rate (ETR) were obtained by increasing PPFD intensity (i.e. 0, 100, 300, 600, 900, 1200, 1500 and 2000 μmol m^-2^ s^-1^) after 30 to 120 s (i.e. instant light curves, [85]). ETR was estimated as:

$$ETR=0.5\times 0.84\times (\Delta F/F_{m}^{´})\times PPDFD$$

where 0.5 stands for an equal distribution of excitation energy to both photosystems II and I and 0.84 for an assumed average light reflection of 16%. The latter measurements were carried out in 1–3 individuals located in 2–3 selected grasslands per species and season. Light-response curves allow the derivation of a number of parameters such as apparent maximal electron transport rate (ETR_max_), PPDF at saturation of photosynthesis (PPDF_sat_) or ΔF/F_m_′ at PPDF_sat_, which reflect the potential intrinsic capacity of leaves and physiological plant plasticity [85]. To estimate ETR_max_ and PPDF_sat_, an exponential rise to maximum function was fitted to the ETR-PPFD curve: y = ae ^(-bx)^, where y is ETR, x is PPFD, and a and b are fitted coefficients. From the latter equation, ETR_max_ was determined as a and PPDF_sat_ as 0.9 ETR_max_ [85]. Finally, ΔF/F_m_′ at PPDF_sat_ was derived from the exponential decay function (y = a (1- e^(-bx)^) fitted to the ΔF/F_m_′ - light response curve, where y is ΔF/F_m_′ and x is PPFD. Following the ecophysiological measurements, the leaves were collected and oven dried at 70°C to constant weight.

### Carbon and nitrogen isotope composition

The oven dried leaves and AGB samples were finely ground with a Vibrator Disc Mill RS 200 and a Mixer Mill MM 200 (Retsch GmbH Haan, Germany). About 1.5 mg of the powdered material from each sample was placed into a tin (Sn) capsule (IVA Analysentechnik, Meerbusch, Germany) for analysis of carbon isotope composition (δ^13^C, ‰) nitrogen isotope composition (δ^15^N, ‰), total nitrogen content (N%, %) and total carbon content (C%, %). The analyses were performed at the ZALF Stable Isotope Facility, Müncheberg, Germany. C%, N%, δ^13^C and δ^15^N were determined using a Flash 2000 HT Elemental Analyzer (Thermo Fisher Scientific, Bremen, Germany) coupled to a Delta V isotope ratio mass spectrometer (IRMS) via a ConFlo IV interface (both Thermo Fisher Scientific, Bremen, Germany). In the case of the soil samples extracted following AGB collection, 20 mg were placed into a tin (Sn) capsule (Säntis Analytical AG, Teufel, Switzerland). The analyses were performed at the WSL Stable Isotope Research Centre (SIRC), Birmensdorf, Switzerland. C%, N%, δ^13^C and δ^15^N were determined using an EA1110 Elemental Analyzer (CE Instruments, Milan, Italy) coupled to a Delta Plus XL ratio mass spectrometer via a ConFlo II interface (both Thermo Fisher Scientific, Bremen, Germany).

Reported isotope ratios were calculated as:

$$\delta^{13}C\;or\;\delta^{15}N\;(‰)=\left(\frac{R_{sample}}{R_{reference}}-1\right)$$

where R_sample_ is the isotopic ratio (^13^C/^12^C or ^15^N/^14^N) of the sample and R_reference_ is the known isotopic ratio of the standard [86]. δ (‰) were referenced against N_2_ in air for δ^15^N and to Vienna Pee Dee Belemnite (VPDB) for δ^13^C. Precision, defined as the standard deviation (±1σ) of the laboratory control standard along the run was better than ±0.1% and ±0.3% for δ^13^C and δ^15^N, respectively.

The average δ^13^C and δ^15^N of each plant group in a given plot (δ^13^C_FGi_ and δ^15^N_FGi_ by functional group, biogeographic status or by their combination) was calculated as the C-weighted and N-weighted average of every replicate in the plot, respectively. In order to estimate the average δ^13^C and δ^15^N at the community level (δ^13^C_C_ and δ^15^N_C_) we applied a stable isotope mixing model were δ^13^C and δ^15^N of each plant group was scaled by the group specific contribution to the total C and N pool. We thus assumed that the contribution of each plant group to δ^13^C_C_ and δ^15^N_C_ was proportional to the relative contribution of that particular group to the total community C pool and N pool, respectively, as described by [87]. That is, average δ^13^C_C_ and δ^15^N_C_ were calculated as the C-weighted and N-weighted average, respectively, of all the plant groups present in a plot:

$$\delta^{13}C_{C}=\sum C_{FGi}\times (\delta^{13}C_{C}/\sum C_{C})\;and\;\delta^{15}N_{C}=\sum N_{FGi}\times (\delta^{13}N_{C}/\sum N_{c})$$

where C_FGi_ and N_FGi_ is the C and N weight (mg) of a particular plant group (either functional or concerning biogeographic status) and C_C_ and N_C_ is the C and N weight of the whole plant community. It should be noted that the C4 species were excluded from the C-weighted average δ^13^C-group calculations as δ^13^C of C4 plants is not indicative of their water use efficiency [88]. However, the abundance of C4 plants in our study sites was very low. The C4 graminoids *Eragostris minor* and *Digitaria* sp. were found in 2 and 1 plots, respectively, where they contributed 10% to the plot community C pool. Their contribution to the total C collected across the study sites was 0.006%. Finally, we calculated the average δ^13^C and δ^15^N of the two selected species in each plot (δ^13^C_Spi_ and δ^15^NS_Spi_).

As a next step, carbon isotope discrimination, which is the carbon isotopic ratio in plant tissue relative to that of the atmosphere, was calculated at the plant group, community and species level as:

$$\Delta^{13}C_{FGi}=\frac{\left(\delta^{13}C_{air}-\delta^{13}C_{FGi}\right)}{\left(1+(\delta^{13}C_{FGi}/1000)\right)}\;\Delta^{13}C_{C}=\frac{\left(\delta^{13}C_{air}-\delta^{13}C_{C}\right)}{\left(1+(\delta^{13}C_{C}/1000)\right)}$$

$$\Delta^{13}C_{{Sp}_{i}}=\frac{\left(\delta^{13}C_{air}-\delta^{13}C_{{Sp}_{i}}\right)}{\left(1+(\delta^{13}C_{{Sp}_{i}}/1000)\right)}$$

where δ^13^C_air_, the ^13^C composition of atmospheric CO_2_, is assumed to be −8 ‰ [88]. Thereafter, intrinsic water use efficiency (iWUE, μmol mol^-1^), that is, the ratio of photosynthetic net CO_2_ assimilation to water loss through stomatal conductance [89], was calculated at the plant group (iWUE_FGi_), community (iWUE_C_) and species level (iWUE_Spi_) by applying the linear model described by [90]:

$$iWUE_{FGi}=\frac{A}{g_{SW}}=\frac{C_{a}}{1.6}\times \left(\frac{b^{´}-\Delta^{13}C_{FGi}}{b^{´}-a}\right)\;and\;iWUE_{C}=\frac{A}{g_{SW}}=\frac{C_{a}}{1.6}\times \left(\frac{b^{´}-\Delta^{13}C_{C}}{b^{´}-a}\right)$$

$$iWUE_{{Sp}_{i}}=\frac{A}{g_{SW}}=\frac{C_{a}}{1.6}\times \left(\frac{b^{´}-\Delta^{13}C_{{Sp}_{i}}}{b^{´}-a}\right)$$

where *A* is net assimilation, g_sw_ is stomatal conductance for water vapor, *C*_*a*_ is the CO_2_ atmospheric mole fraction (400 μmol mol^-1^ CO_2_, [91]), *a* is the fractionation during CO_2_ diffusion through the stomata (4.4‰, [92]), and *b´* is the fractionation associated to the Rubisco and PEP carboxylase reactions (27‰, [93]).

To correct δ^15^N values for site-specific differences in background bulk soil δ^15^N, we estimated the ^15^N enrichment factor of biomass compared to soil background values [94] at the community (Δδ^15^N_C_), plant group (Δδ^15^N_FGi_) and species level (Δδ^15^N_Spi_) as:

$${\Delta \delta }^{15}N_{FGi}=\delta^{15}N_{FGi}-\delta^{15}N_{Soil}\;and\;{\Delta \delta }^{15}N_{C}=\delta^{15}N_{C}-\delta^{15}N_{Soil}$$

$${\Delta \delta }^{15}N_{Spi}=\delta^{15}N_{Spi}-\delta^{15}N_{Soil}$$

Δδ^15^N can be interpreted as an indicator of cumulative N losses, i.e. due to the discrimination of gaseous and hydrological N export processes against ^15^N, higher Δδ^15^N values indicate “open N cycles” with important N losses, while lower values indicated “closed N cycles” [95].

### Data analysis

The relationship between species richness and AGB, iWUE or Δδ^15^N was assessed by regression analysis. To test if the relationship between biodiversity and AGB in Berlin grasslands was comparable to that found in multiple experimental grasslands across Europe, we extracted numerical data from plots in Fig 3 in [36] using Web Plot Digitizer v4.1 (https://apps.automeris.io/wpd/). To ensure that differences in the biodiversity-AGB relationship were not stemming from environmental differences among grassland locations, the extracted data were min-max normalized. The slopes from the corresponding biodiversity-AGB significant regression or ANOVA models were then synthesized.

Random forest (RF) analyses was used to identify the main factors driving the variability in AGB, iWUE and Δδ^15^N across the studied urban grasslands in Berlin at the plant group (i.e. concerning functional group or biogeographic status) and community level and to thereby assess the relative contribution of biodiversity, biotic and abiotic novelty in controlling urban grassland functioning. These factors included functional and taxonomic diversity, the BNI, environmental variables related to abiotic novelty but also moss and litter cover in the grasslands. We used a conditional inference random forest algorithm [96, 97] to deal with missing values, nonlinear associations among variables, and a number of predictors larger than the number of samples. RF was applied for each response variable to estimate the relative importance of the predictors, measured as the contribution to model fitting accuracy of R^2^ with variable selection [98]. RF can robustly estimate the relative importance among highly correlated predictors (e.g. land use cover proportion at different buffer radii; [99–101]. As small sample size can result in instable estimates [102], we took the following strategies. Firstly, we determined the number of tree hyper-parameter (ntree = 2000) after confirming its performance stability compared with lower numbers (10, 100, and 1000). Secondly, we estimated variable importance 100 times with changing random seed for randomization and then took an average. Finally, we estimated statistical significance for each predictor, i.e. the probability that the averaged importance score is obtained just by chance (999 permutation; alpha = 0.05) [102, 103]. Thereby, significant predictors were selected and their explanatory power (R^2^) was estimated for each response variable. For non-significant predictors, variable importance was set to 0 [103]. Modeled association patterns between significant predictors and each response variable were visualized using partial dependence plot (PDP) approach, which averages out the other predictors’ effects to confirm the direction of the effects but not the effect size [104]. All procedures were done in R v3.5.1 [105], with ‘party’ and ‘mlr’ packages [106, 107]. The R script including the other hyperparameter values is available at [108].

It was not possible to obtain a RF model for every plant group (an overview of the RF models developed is provided in S2 Table). This was partly due to the sparse presence of some functional groups at the plots (i.e. alien graminoids and alien legumes), but also due to a relatively limited sample size and the lack of explanatory power of the predictors; we emphasize, however, that the sample size (i.e. the number of sites) was relatively large for such a field study, where all plots had to be sampled and measured within a short time frame for comparability. Likewise, the available data was not sufficient to identify the main factors driving the variability in photosynthetic parameters, iWUE and Δδ^15^N at the species level by using RF model analysis. Therefore, we opted for analyzing the correlation (Spearman´s rho, r_s_) between AGB, iWUE or Δδ^15^N at the species level and species richness, SVF or BNI as representative parameters for biodiversity, abiotic and biotic novelty, respectively. Mann-Whitney or Kruskal-Wallis with pairwise multiple comparison tests were used to analyze significant differences (p < 0.05) in iWUE and Δδ^15^N among plants with different biogeographic status (i.e. native *vs* alien) or plant from different functional groups. The Mann-Whitney test was also used to analyze seasonal differences in photosynthetic parameters, iWUE and Δδ^15^N at the species level. The aforementioned analyses were performed with IBM^®^ SPSS^®^ Statistics 22.0.0.0 Software.

## Results

### Variability in aboveground biomass, intrinsic water use efficiency and ^15^N enrichment factor

**Fig 2:**
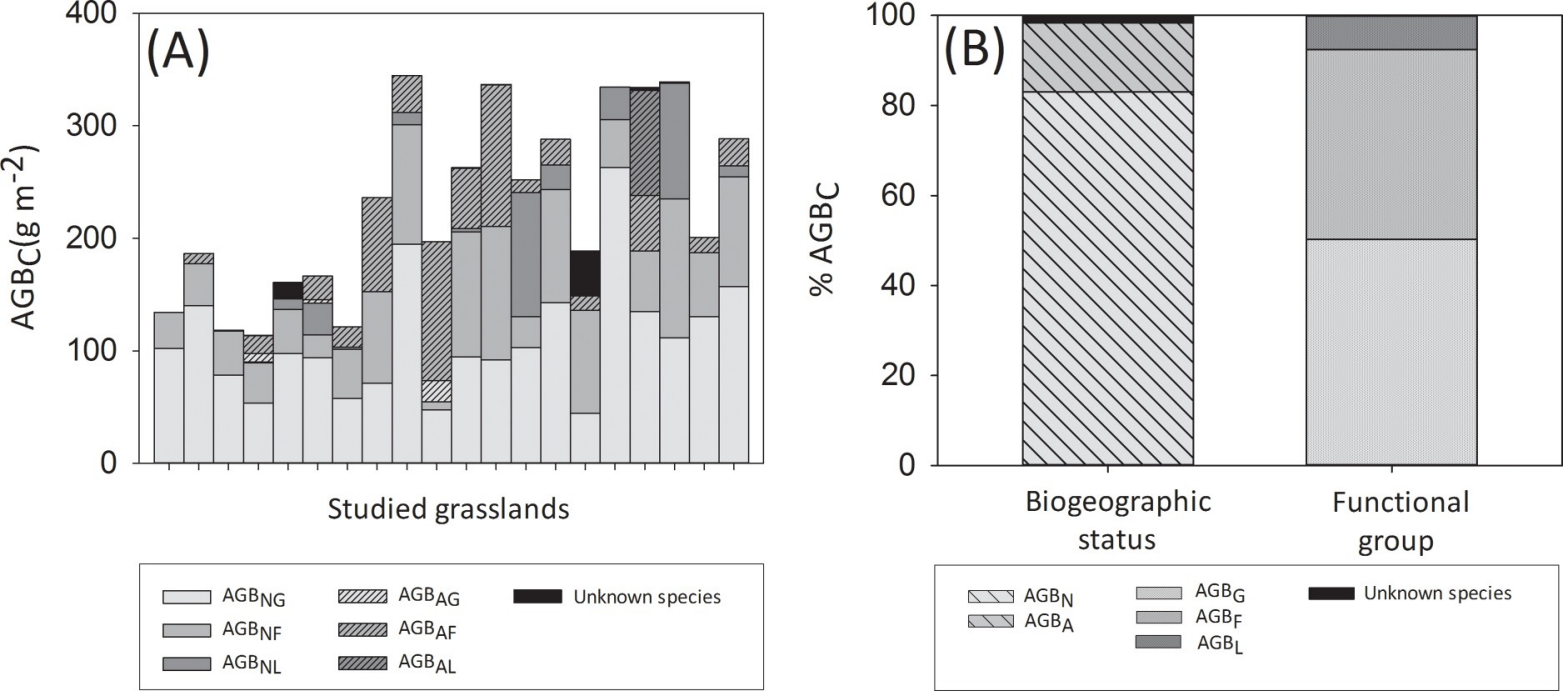
Contribution of plant groups to grassland community above ground biomass. (A) Average contribution of plant functional groups with different biogeographic status to community aboveground biomass in the 20 studied grasslands (AGB_C_), where grasslands are ordered from left to right according to increasing species richness and (B) average percentage contribution of plant functional groups and plants with different biogeographic (i.e. graminoids (AGB_G_), forbs (AGB_F_) and legumes (AGB_L_), natives (AGB_N_), aliens (AGB_A_), native graminoids (AGB_NG_), alien graminoids (AGB_AG_), native forbs (AGB_NF_), alien forbs (AGB_AF_), native legumes (AGB_NL_), alien legumes (AGB_AL_)) to AGB_C_ across the studied grasslands in Berlin in summer 2017.

**Fig 3:**
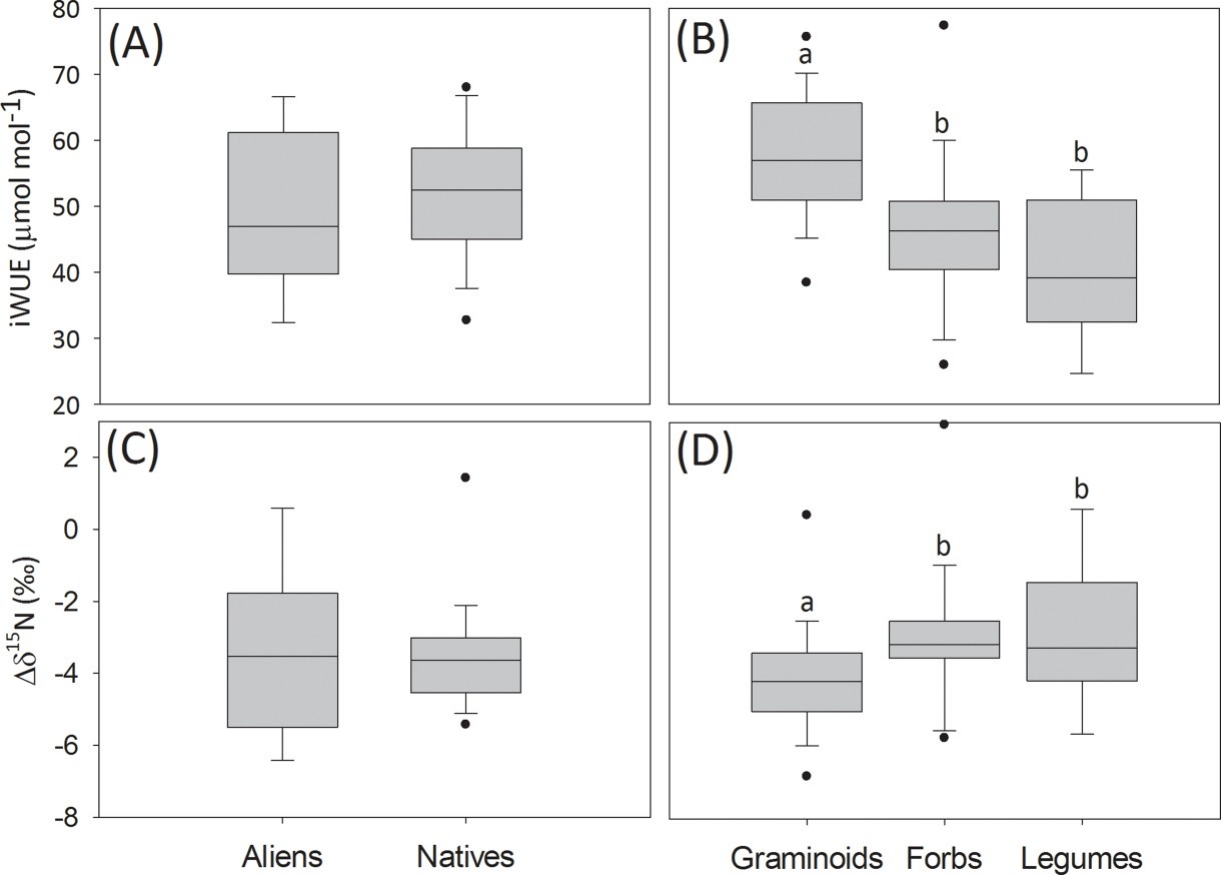
Intrinsic water use efficiency and ^15^N enrichment factor of different plant groups. (A) Average intrinsic water use efficiency (iWUE) of plants with different biogeographic status and (B) plants belonging to different functional groups; (C) average ^15^N enrichment factor (Δδ^15^N) of plants with different biogeographic status and (D) plants belonging to different functional groups across the studied grasslands in Berlin in summer 2017. Box plots indicate interquartile ranges (areas within a box), medians (horizontal line within the box), 25th and 75th percentiles (lower and upper box boundaries), and 5th and 95th percentiles (whiskers above and below the box); outliers are shown as solid circles. Significant differences between plant with different biogeographic status (p < 0.05, Mann-Whitney test) or functional groups (p < 0.05, Kruskal-Wallis test with pairwise multiple comparisons) are indicated by letters.

The 20 urban grasslands studied in Berlin covered a wide range of plant species richness and functional diversity as well as biotic and abiotic novelty (Table 1). Plant species richness varied more than threefold among grasslands and the BNI (biotic novelty index) showed a tenfold variation. Likewise, soil parameters, climate and urban parameters showed wide value ranges across sites. Total plant community aboveground biomass (AGB_C_) varied threefold across the studied urban grasslands in late summer 2017 (Fig 2A). Important variations were also found between replicates within plots in AGB_C_ (CV = 5–66%, mean 30%). Overall, native species dominated AGB_C_, contributing more than 60% in 18 out of 20 plots (Fig 2B). Graminoids and forbs made up for most of AGB_C_ (on average >90%) and their share was similar, whereas legumes contribution was relatively small (Fig 2B). All legume and graminoid species present in the sampled quadrats were native to Berlin with the exception of *Medicago x varia* T. Martyn and *Eragrostis minor* Host, which were found in one and two plots, respectively. Indeed, the average contribution of alien legumes and graminoids to AGB_C_ across the 20 studied urban grasslands was 1.4% and 0.9%, respectively. However, approximately one third of the forb species were alien to Berlin, and these were present in most plots, contributing on average 13.1% to AGB_C_ in the study sites. An overview of the species present in the sampled quadrats is provided in S3 Table. Both iWUE_C_ and Δδ^15^N_C_ varied markedly among urban grasslands, having an average value of 50.84 μmol mol^-1^ (SE = 0.44) and -3.43 ‰ (SE = 0.37), respectively. Plants with different biogeographic status did not significantly differ in their iWUE or Δδ^15^N (Mann-Whitney, p > 0.05; Fig 3A and 3B). However, iWUE_G_ was significantly higher than iWUE_F_ and iWUE_L_ (Kruskall-Wallis, p < 0.05; Fig 3C) and Δδ^15^N_G_ was significantly lower than Δδ^15^N_F_ and Δδ^15^N_L_ (Kruskall-Wallis, p < 0.05; Fig 3D).

**Fig 4:**
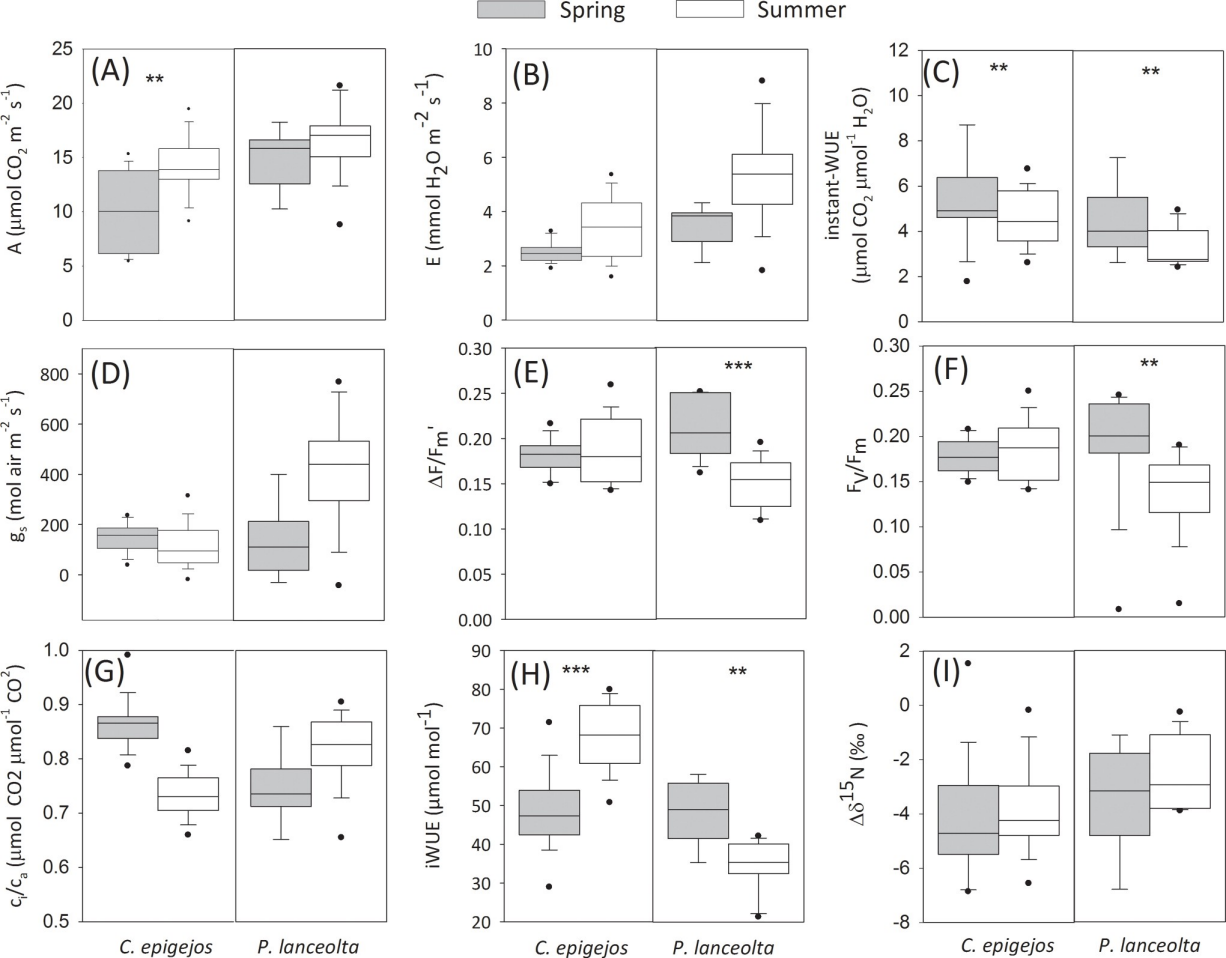
Seasonal difference in ecophysiological parameters at the species level. (A) Photosynthetic rate (A), (B) transpiration rate (E), (C) instantaneous water-use efficiency (instant-WUE), (D) stomatal conductance (g_s_), (E) effective quantum yield of electron transport through photosystem II (ΔF/F_m_′), (F) maximum potential quantum yield of electron transport through photosystem II (F_v_/F_m_), (G) ratio of the intercellular to ambient CO_2_ concentration (c_i_/c_a_), (H) Intrinsic water use efficiency (iWUE) and (I) ^15^N enrichment factor (Δδ^15^N) in spring (grey) and summer (white) 2017. Box plots indicate interquartile ranges (areas within a box), medians (horizontal line within the box), 25th and 75th percentiles (lower and upper box boundaries), and 5th and 95th percentiles (whiskers above and below the box). Significant differences between seasons (p < 0.05, Mann-Whitney test) are indicated by asterisks: ** p<0.01 and *** p<0.001).

**Fig 5:**
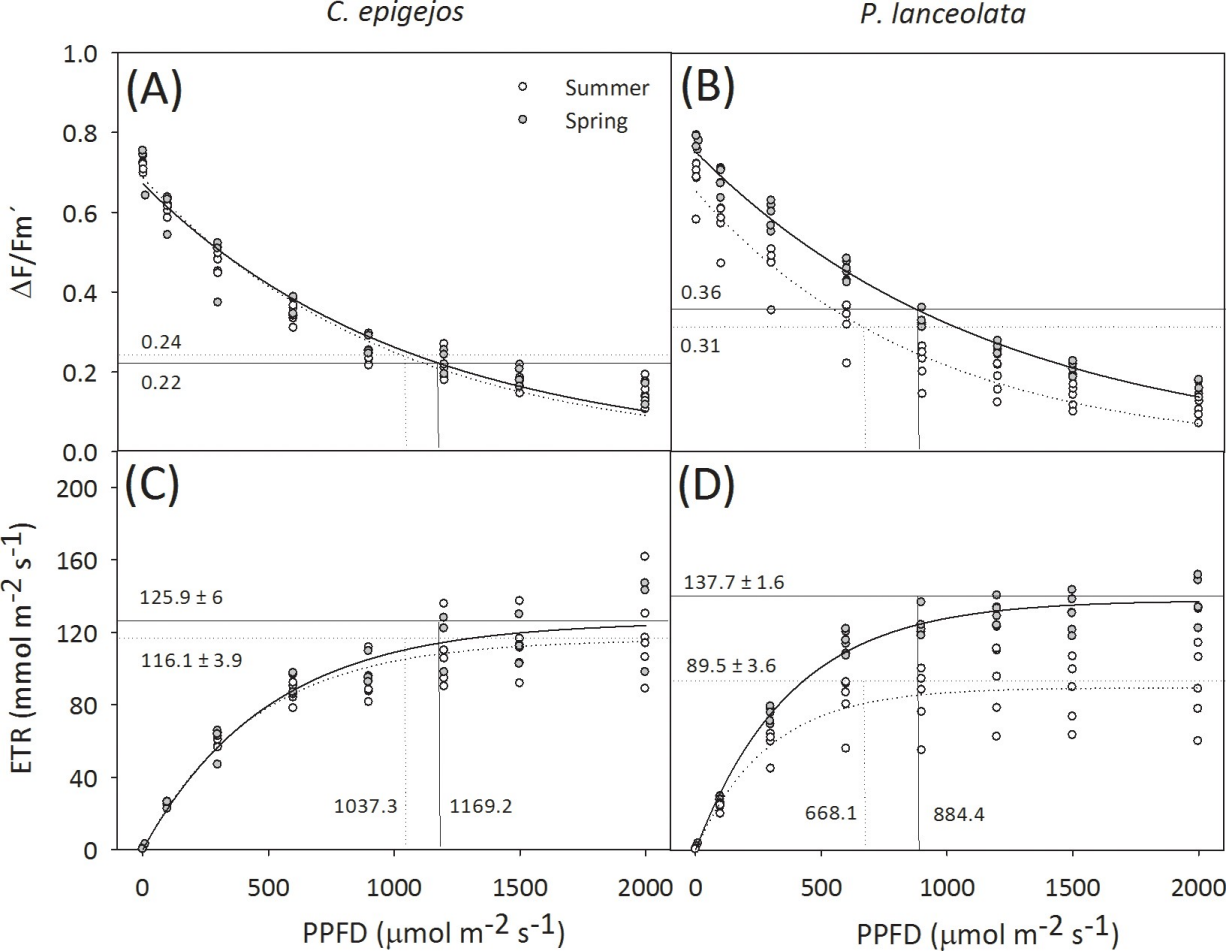
Photosynthetic light response curves. (A) Effective quantum yield of electron transport through photosystem II, ΔF/F_m_′ in *Calamagrostis epigejos* and (B) *Plantago lanceolata*; (C) apparent photosynthetic electron transport rate, ETR in *C*. *epigejos* and (D) *Pl*. *lanceolata*. Shown data are single values from chlorophyll fluorescence measurements on 3 to 9 individuals in spring (solid lines) and summer (dotted lines) in Berlin grasslands in 2017. Regression lines were fitted with an exponential decay function for ΔF/F_m_′ (y = a (1- e^(-bx)^) (A, B) and with an exponential rise to max function for ETR: y = ae ^(-bx)^ (C, D). The numbers at the horizontal and vertical lines in the lower panels indicate apparent maximal electron transport rate (ETR_max_) and PPDF at saturation of photosynthesis (PPDF_sat_), respectively. Numbers at the horizontal lines in the upper panels indicate ΔF/F_m_′ at PPDF_sat_.

With regard to the seasonal differences at the species level, in *C*. *epigejos* A increased significantly from spring to summer, whereas instant-WUE decreased (Mann-Whitney, p > 0.05; Fig 4A and 4C). In *P*. *lanceolata*, both instant-WUE, F_v_/F_m_ and ΔF/Fm′ significantly decreased from spring to summer (Mann-Whitney, p > 0.05; Fig 4C, 4E and 4F). Whereas iWUE_C.ep_ significantly increased from spring to summer, iWUE_P.la_ significantly decreased over the growing season but no seasonal differences were found for Δδ^15^N_C.ep_ or Δδ^15^N_P.la_ (Fig 4H and 4I). Intrinsic photosynthetic capacity, as indicated by the light-response curves and their associated cardinal points, did not show important seasonal variations in the case of *C*.*epigejos* but decreased from spring to summer in *P*.*lanceolata* (Fig 5).

### Key predictors for the functioning of urban grasslands

**Fig 6:**
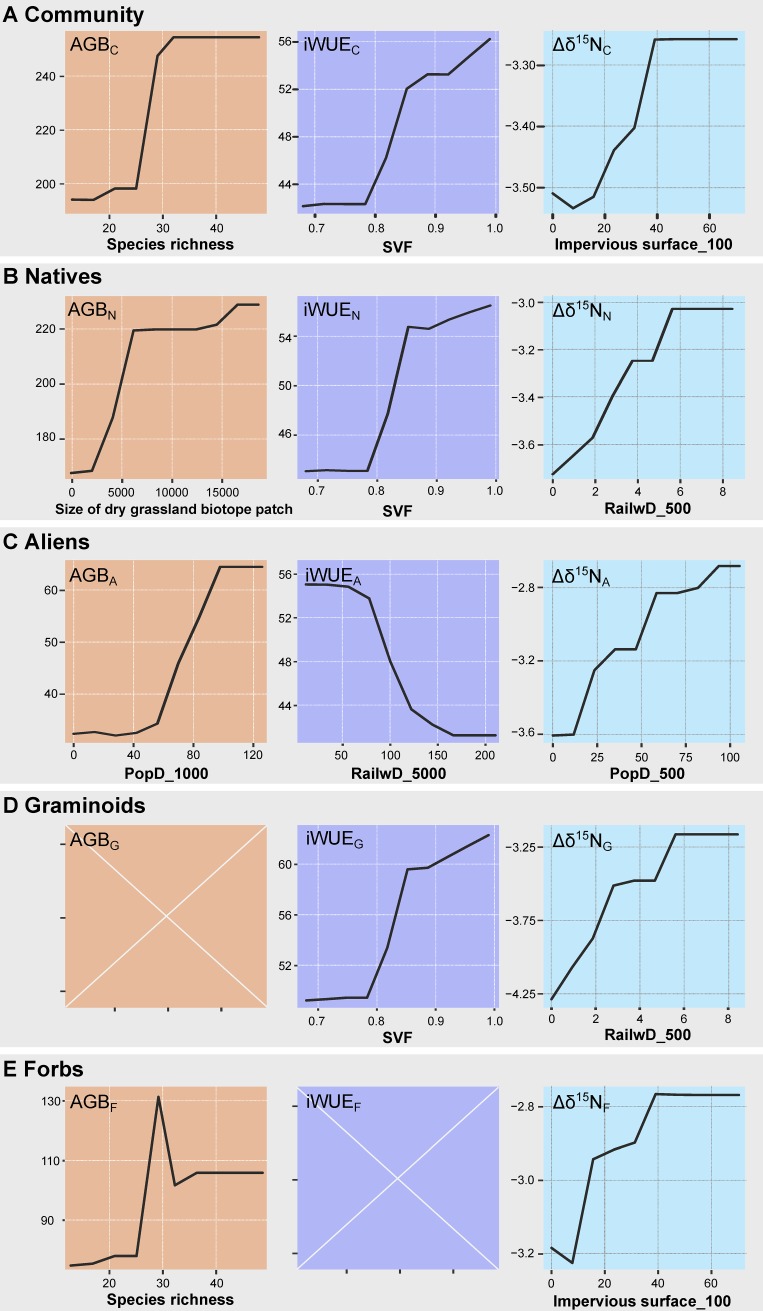
Partial dependence plots for the strongest predictor selected by random forest models for aboveground biomass, intrinsic water use efficiency and ^15^N enrichment factor. (A) for the whole plant community (AGB_C_, iWUE_C_, Δδ^15^N_C_), (B) natives (AGB_N_, iWUE_N_, Δδ^15^N_N_), (C) aliens (AGB_A_, iWUE_A_, Δδ^15^N_A_), (D) graminoids (AGB_G_, iWUE_G_, Δδ^15^N_G_) and (E) forbs (AGB_F_, iWUE_F_, Δδ^15^N_F_) in the studied grasslands in Berlin in summer 2017. The full description of variables is given in Table 1. Note that a partial dependency plot is used not to confirm the effect size but the association pattern including effect direction, since due to normalization, the ranges of y-axes do not directly correspond to the range of the variable. Plots for AGB_G_ and iWUE_F_ were not computed either because only one predictor was selected or because of lack of explanatory power (see text for more information).

In the following, we report the RF models´ results obtained for AGB, iWUE and Δδ^15^N. For each ecosystem functioning proxy, we first described the RF models´ results for the whole plant community, followed by those for plant biogeographic status classes, plant functional groups and finally for the combination of the latter two. As mentioned above, it was not possible to obtain a RF model for every plant group. Therefore, the RF model description is not consistent among ecosystem functioning proxies and plant groups and we decided to only include results of those plant groups that were more consistently modelled in Fig 6. The results for all the plant groups are provided in the S1 File, S2 File and S3 File.

#### Aboveground biomass

The explanatory power (R^2^) of the AGB_C_, AGB_N_ and AGB_A_ random forest models were 0.87, 0.83 and 0.76, respectively, with a corresponding validation accuracy (R^2^) of 0.36, 0.41 and 0.17. Out of 27 explanatory variables, six were selected for the AGB_C_ RF model. Species richness was the most important predictor (R^2^contribution: 41%), followed by size of dry grassland biotope patch (16%), moss cover (12%), functional diversity (8%), SVF (7%) and litter cover (3%). While AGB_C_ was predicted to markedly increase above 25 plant species and a dry grassland biotope patch size of 3500 m^2^, AGB_C_ diminished with increasing moss cover, being this trend especially strong at moss cover < 10% (Fig 6A; Figure A in S1 File). The five variables selected for the AGB_N_ RF model partially overlapped with those selected for the AGB_C_ and included in descending order of importance: size patch, litter cover, relative humidity, species richness and N soil content. As for the AGB_C_ RF model, the AGB_N_ RF model predicted a monotonic increase with species richness (8%), size of dry grassland biotope patch (40%) and litter cover (22%; Fig 6B; Figure B in S1 File). In the case of AGB_A_, most predictors selected for the RF model were related to abiotic novelty and specifically to urban parameters. In general terms, AGB_A_ increased monotonically with both PopD and FAR in buffer areas of multiple sizes around the biotope patch (R^2^: 59% and 11%, respectively; Fig 6C; Figure C in S1 File). Functional diversity influenced AGB_A_ positively as well (Figure C in S1 File), but its explanatory power was low (6%).

The explanatory power (R^2^) of the AGB_G_ and AGB_F_ RF models was 0.57 and 0.72, with a corresponding validation accuracy of 0.17 and 0.30. AGB_G_ decreased with increasing moss cover, which was the only predictor selected for the AGB_G_ RF model (R^2^ contribution: 57%; Fig 6D). Species richness was the main predictor for AGB_F_ (53%), followed by functional diversity (17%) and litter cover (3%). For the two strongest predictors, the RF model revealed a hump-shaped relationship (Fig 6E; Figure D in S1 File). AGB_NG_, AGB_NF_ and AGB_AF_ revealed a similar pattern to that observed for the overall plant groups with different biogeographic status (Figures E-F in S1 File). AGB_NG_ showed a strong monotonic negative dependence on moss cover (61%; Figure E in S1 File). Whereas the size of dry grassland biotope patch and species richness were the main predictors for AGB_NF_ (55% and 10%, respectively), abiotic novelty-related variables explained the variance observed in AGB_AF_ (Figures F-G in S1 File). Specifically, AGB_AF_ increased monotonically with PopD in buffer areas of multiple sizes around the biotope patch (39%) but declined at low Pb (11%).

#### Intrinsic water use efficiency

The explanatory power (R^2^) of the iWUE_C_, iWUE_N_ and iWUE_A_ RF models was 0.84, 0.85 and 0.88, respectively, with a corresponding validation accuracy of 0.50, 0.56 and 0.45. A total of 4 variables were selected for the iWUE_C_ RF model. SVF was the most important predictor (R^2^ contribution: 62%), followed by Wc, share of grassland and Cu (8%, 7% and 6%, respectively). Community iWUE increased monotonically above a SVF of 0.8 and showed a rapid increase when the share of grassland was very low, reaching a maximum at ≈ 5% (Fig 6A; Figure A in S2 File). Likewise, iWUE_C_ varied markedly at the lower Cu and Wc ranges, but in this case its response was negative (Figure A in S2 File). The four predictors selected for the iWUE_N_ mostly overlapped with those selected for iWUE_C_, with SVF being also the strongest predictor for iWUE_N_ (53%) and having a positive effect (Fig 6B; Figure B in S2 File). Additional parameters such as Wc, TN and Cu content accounted for 30% of R^2^ and showed a negative e relation with iWUE_N_ (Figure B in S2 File). RailwD_5000 (47%) was the main predictor for iWUE_A_, although SVF and AirT were also relevant for (26% and 15%, respectively). iWUE_A_ decreased monotonically with increasing RailwD_5000 but showed a positive response to SVF and AirT (Fig 6C; Figure C in S2 File). The explanatory power (R^2^) of the iWUE_G_ RF model was 0.83, with a validation accuracy of 0.49. iWUE_G_ increased with increasing SVF (49%), but decreased with Zn, W_C_ and Cu (14%, 13% and 8%, respectively; Fig 6; Figure D in S2 File). The iWUE_NG_ and iWUE_AF_ RF models revealed a similar pattern to that observed for the overall plant groups with different biogeographic status (Figures E-F in S2 File). iWUE_NG_ increased monotonically with SVF (39%) but decreased with W_C_ and N (14% and 10%, respectively. iWUE_AF_ decrease with increasing RailwD_5000 (53%) and showed significantly lower values in new plots (10%) (Figures E-F in S2 File).

#### ^15^N enrichment factor

The explanatory power (R^2^) of the Δδ^15^N_C_, Δδ^15^N_N_ and Δδ^15^N_A_ RF models was 0.71, 0.7 and 0.75, respectively, with a corresponding validation accuracy of 0.2, 0.22 and 0.3. The 8 predictors selected for the Δδ^15^N_C_ RF model in descending order of importance when summing up the explanatory power of the predictors for buffer areas of multiple sizes around the biotope patch were FAR (R^2^ = 0.17), RailwD (14%), PopD (14%), impervious surface (12%) and share of grassland (7%), followed by UrbClim (3%), RoadD_100 (2%) and AirRH (2%) (Fig 6A; Figure A in S3 File). Note that when considering separately the explanatory power of the predictors for different buffer areas sizes around the biotope patch, impervious surface_100 was the main predictor selected by the model. The predictors selected for Δδ^15^N_N_ and Δδ^15^N_A_ mostly overlapped with those selected for Δδ^15^N_C_, though their explanatory power differed (Figures A-C in S3 File). The main predictor for Δδ^15^N_N_ was RailwD in buffer areas of multiple sizes (34%), followed by impervious surface_100 (14%), share of grassland_5000 (9%), FAR in buffer areas of multiple sizes (8%), PopD_500 (3%) and AirRH (3%) (Fig 6B; Figure B in S3 File). However, PopD and FAR in buffer areas of multiple sizes (32% and 22%) were the strongest predictors for Δδ^15^N_A_, followed by impervious surface, AirT_long term, UrbClim and share of grassland 500 (8%, 8%, 3% and 2%, Fig 6C; Figure C in S3 File)). All the aforementioned predictors showed a positive monotonic relationship with Δδ^15^N_C_, Δδ^15^N_N_ and Δδ^15^N_A_ except for share of grassland, AirRH and UrbClim, which showed an overall negative relationship with the response variables (Fig 6A–6C; Figures A-C in S3 File). The explanatory power (R^2^) of the Δδ^15^N_G_ and Δδ^15^N_F_ RF models was 0.45 and 0.21, with a corresponding validation accuracy of 0.14 and 0.17. Δδ^15^N_G_ increased monotonically with RailwD in buffer areas of multiple sizes around the biotope patch and impervious surface_100 (45% and 14%) but decreased with share of grassland_5000 (13%) and similarly, Δδ^15^N_F_ RF increased with FAR, PopD, impervious surfaceand RailwD when summing up the explanatory power of the predictors for buffer areas of multiple sizes around the biotope patch (21%, 17%, 16% and 13%; Figures D-E in S3 File). Note that when considering separately the explanatory power of the predictors for different buffer areas sizes around the biotope patch, impervious surface_100 was the main predictor selected for Δδ^15^N_F_ RF model. The Δδ^15^N_NG_, Δδ^15^N_NF_ and Δδ^15^N_AF_ RF models reflected the pattern observed for the overall plant groups with different biogeographic status, with RailwD, FAR, impervious surface and PopD in buffer areas of multiple sizes around the biotope patch being the main predictors (Figures F-H in S3 File).

### Biodiversity-ecosystem functioning in urban grasslands

**Fig 7:**
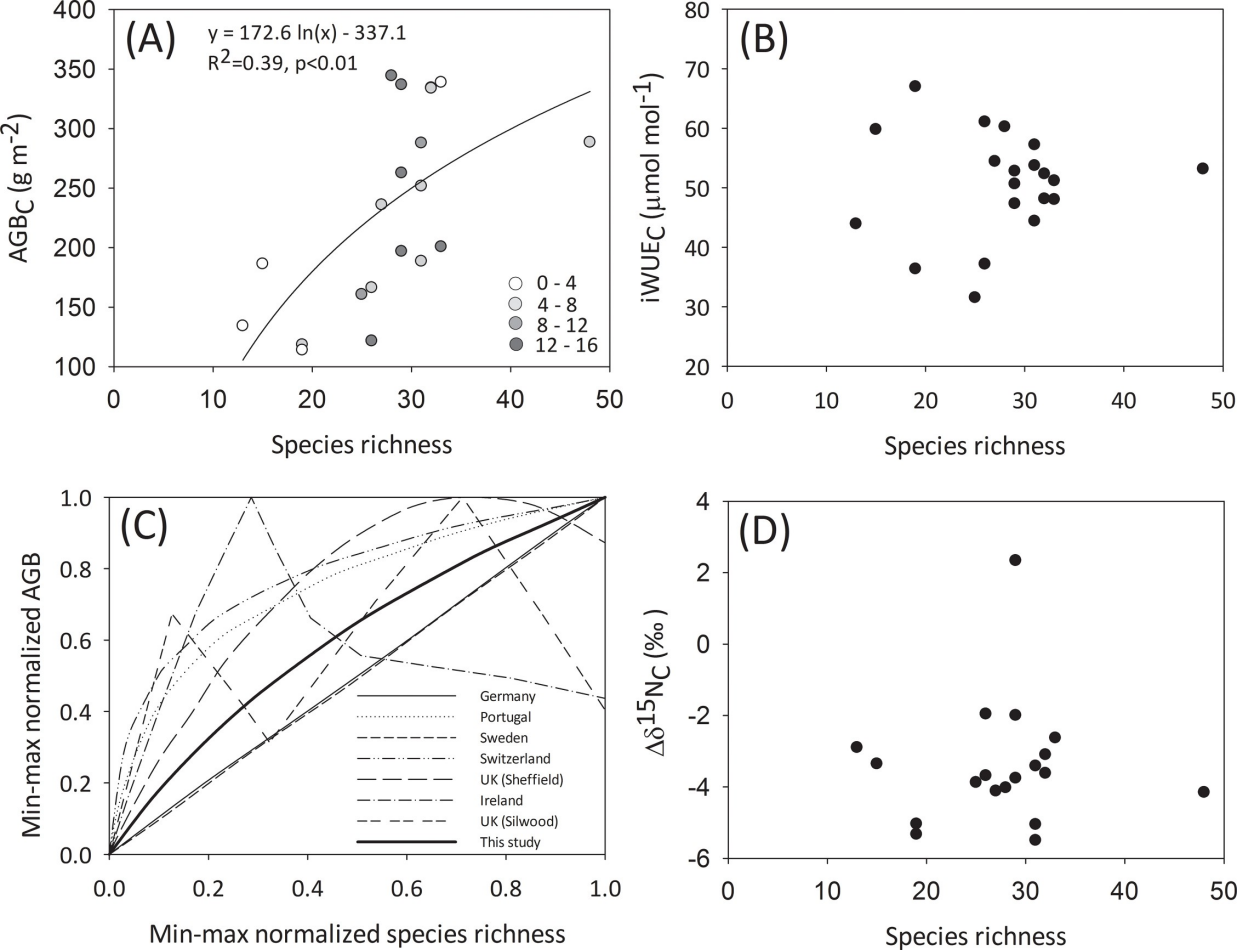
Biodiversity-ecosystem functioning in urban grasslands. Relationship between species richness and (A) community aboveground biomass (AGBC), where color scale indicates the percentage of aliens in each plot, (B) community intrinsic water use efficiency (iWUEC) and (D) community ^15^N enrichment factor (Δδ^15^N_C_) in Berlin grasslands in summer 2017. Given values are the average at each grassland. (C) Slopes of significant regression models reflecting the relationship between min-max normalized species richness and min-max normalized AGBC in Berlin novel grasslands in summer 2017 and in multiple European experimental grasslands (extracted from (36), S1 Dataset).

AGB_C_ increased significantly with species richness in urban grasslands (Fig 7A). Namely, AGB_C_ increased up to a saturating value of 350 g m^-2^ at 30 species. The relationship between species richness and AGB_C_ was not influenced by the proportion of alien species (Fig 7A). The positive biodiversity-aboveground biomass relationship in non-manipulated urban grasslands was similar to that found in experimental non-urban grasslands across Europe, in other words, the regression slope in our study fell within the slope range reported by [36], Fig 7C. Unlike in the case of AGB_C_, species richness was not significantly correlated to iWUE_C_ or Δδ^15^N_C_ (Fig 7B and 7D).

### Species level AGB, iWUE or Δδ^15^N

Species richness was not significantly correlated with photosynthetic parameters, iWUE or Δδ^15^N at the species level. The BNI did not affect photosynthetic parameters of *C*. *epigejos* and *P*. *lanceolata*, iWUE_C.ep_ or iWUE_P.la_ but it was negatively correlated to Δδ^15^N_C.ep_ in summer. In *C*. *epigejos*, both A (r_s_ = 0.59, p = 0.036, n = 13) and g_s_ (r_s_ = 0.61, p = 0.028, n = 13) increased with SVF in spring. No significant correlation was found between SVF and photosynthetic parameters in *C*.*epigejos* in spring or in *P*.*lanceolata* in spring and summer. iWUE_C.ep_ increased significantly with SVF in both spring and summer (r_s_ = 0.8, p = 0.001, n = 13; r_s_ = 0.53, p = 0.029, n = 17). No effect of SVF on Δδ^15^N_C.ep_ or Δδ^15^N_P.la_ was found.

## Discussion

The relationship between biodiversity and ecosystem function has received much attention during the last decades in ecological research (e.g. [34, 36, 39, 109]). At present, accelerated urban sprawl poses the question whether urban ecosystems are able to provide services in a comparable way to their non-urban counterparts. We focused on aboveground biomass, intrinsic water use efficiency and ^15^N enrichment factor (AGB, iWUE and Δδ^15^N) as proxies for biomass production, water and N cycling, respectively, which have been acknowledged as relevant processes for ecosystem integrity [110]. As a major insight we found that biodiversity, abiotic, and biotic novelty were related differently to these ecosystem functions. Community aboveground biomass (AGB_C_) was mainly explained by species richness. In contrast, community intrinsic water use efficiency (iWUE_C_) and community ^15^N enrichment factor (Δδ^15^N_C_) were mostly determined by light availability (depicted by the SVF) and urban parameters, respectively, which overrode biodiversity effects. In addition, our results indicate that abiotic novelty potentially favors alien plants in Berlin, mainly by enhancing their dispersal and fitness (via increased iWUE) under low water availability conditions. We found that abiotic novelty, exemplified by urban parameters, specifically affected aboveground biomass and intrinsic water use efficiency of aliens (AGB_A_ and iWUE_A_), but this effect did not translate into effects on the community level. However, abiotic novelty did affect both ^15^N enrichment factor of natives and aliens (Δδ^15^N_N_ and Δδ^15^N_A_), suggesting a broader impact of urbanization on N cycling compared to C and water cycling. At the species level, abiotic novelty, and specifically sky view factor (SVF), appeared to be the prevailing driver of photosynthetic performance and resource use efficiency over both species richness and biotic novelty.

### Key predictors for the functioning of urban grasslands

#### Aboveground biomass

Plant invasions have been linked to declines in species diversity and increases in primary production, often attributed to the presence of highly productive alien species [47]. In the studied urban grasslands in Berlin, the relationship between species richness and AGB_C_ was not influenced by the proportion of alien species, that is, lower species richness was not necessarily associated to a higher share of aliens. We found that AGB_C_ increased significantly with plant species richness and more importantly, according to the RF models, species richness was the strongest predictor for AGB_C_. Although we did not explicitly assess complementarity and selection effects [111] in our study, the fact that the increase in AGB_C_ was associated with increasing Rao´s Q suggests that complementarity was the main mechanisms driving the observed diversity-ecosystem functioning relationship. Functional diversity quantifies trait variability in a given community, and it has been suggested to foster niche complementarity by reducing niche overlap among species pools comprising higher trait ranges [110]. The positive effect of size of dry grassland biotope patch on AGB_C_ further suggests that biomass is boosted by complementarity effects, since biotope space strengthens the biodiversity-AGB relationship by enabling higher niche complementarity among species [112].

Our results additionally indicate that light availability (depicted by the SVF) limits primary production in urban grasslands in Berlin. Moss cover was also associated to reduced AGB_C_, mainly via a decrease in the aboveground biomass of graminoids (AGB_G_). Numerous studies have described a negative relationship (mostly attributed to competition) between bryophyte and vascular plant biomass [113]. For instance, in the arctic tundra, moss cover was associated to lower graminoids productivity via reduced soil temperature and N availability [114]. However, our results do not provide enough information to elucidate the mechanisms behind this relationship and reduced vascular plant aboveground biomass production might have also relieved light competition for bryophytes.

Our study points to differing controlling factors for the AGB of plants with different biogeographic status. Whereas aboveground biomass of natives (AGB_N_) was mainly affected by plant diversity and size of dry grassland biotope patch, aboveground biomass of aliens (AGB_A_) was mainly explained by abiotic novelty-related parameters, specifically PopD (population density). We hypothesize that this discrepancy stems from different predominant dispersal mechanisms of the alien and native species most frequently found in our plots. The most common aliens were *Berteroa incana* (L.) DC. and *Conyza canadensis* (L.) Cronquist, found in 9 and 4 plots, respectively (the geographic location of these plots is provided in S4 Table). Despite the fact that these species are predominantly wind-dispersed, dispersal via footwear and mowing machines have been reported as potential dispersal mechanisms for *B*. *incana* [115]. Unintended human-mediated dispersal by vehicles [116] or clothing attachment [117] was found to effectively disperse *C*. *canadensis*, which also has highly volatile seeds to the extent that may even reach the planetary boundary layer of the atmosphere [118]. Human-mediated dispersal is known to increase with PopD due to higher density of transport networks integrated by roads or walking routes [119]. By contrast, the most common native species in our plots were the perennial grasses *C*. *epigejos* and *Festuca trachyphylla* (Hack.) Hack (present in 9 and 11 plots, respectively). These clonal species seem to prioritize vegetative over seed reproduction once they are established [120] which reduces the probability of human-mediated dispersal. These results are in line with those from previous studies showing that alien species diversity increased with settlement connectivity and number of inhabitants along an urban-rural gradient in the vicinity of Frankfurt (Germany), which suggests that dispersal mechanisms of alien species are associated with human mobility patterns [121].

#### Intrinsic water use efficiency

Unlike in the case of AGB_C_, species richness was not significantly correlated to iWUE_C_ in the studied urban grasslands. In our study, environmental factors related to resource availability and urbanization seemed to override biodiversity effects. In particular, SVF was the strongest predictor for iWUE_C_ followed by soil gravimetric water content (W_C_). A higher SVF implies higher incoming solar radiation, which very likely boosted non-light-saturated photosynthesis, resulting in increased iWUE_C_ in grasslands with a higher share of open sky. The increasing trend in iWUE_C_ observed with decreasing W_C_ most likely reflected lowered stomatal conductance to avoid water loss associated to increasing water stress [122]. While a positive biodiversity-aboveground biomass relationship seems to be relatively general across ecosystems and studies, and even robust to nutrient enrichment and drought [33], the contrasting patterns how biodiversity relates to water use reported in the literature point to a more variable relationship. For instance, species mixtures were found to be more efficient in water use (estimated via water balance) in comparison with monocultures in experimental grasslands and this was attributed to complementary [50]. By means of a soil water balance modelling approach, a more complete exploitation of soil water, ascribed to higher root density, was linked to higher diversity in experimental grasslands [56]. In contrast, stable water isotopologue labeling revealed no positive effects of species richness on water uptake by plant communities in the temperate experimental grasslands of the Jena Experiment [123].

The differences in iWUE among functional groups is consistent with previous observations in natural grasslands, in which δ^13^C_G_ was lowest, followed by δ^13^C_F_ and δ^13^C_L_ ley [124, 125]. Higher stomatal conductance—which might be a reason for lower iWUE—in forbs compared to grasses was observed in soil-plant monoliths from the Jena experiment and attributed to water uptake from deeper and thus moister soil layers [126]. Even though plants with distinct biogeographic status did not significantly differ with regard to their iWUE, different dominant drivers were identified for natives and aliens. Intrinsic water use efficiency of natives (iWUE_N_) was mainly controlled by light and to a lesser extent by water availability (i.e. SVF and W_C_). However, urbanization, and specifically railway density (RailwD) in a buffer area of 5 km, was the main factor modulating iWUE_A_. Railway embankments functioned as active water sources to nearby ecosystems in a Mediterranean watershed by increasing runoff [127]. This increased water supply could be one reason for aliens spreading along railways [128] and increased iWUE_A_ with decreasing RailwD might point to lower stomatal conductance associated with increasing water stress in areas with less dense railway networks. [129] indicated that aliens often have competitive physiological traits that increase their fitness in unfavorable environments, including the capacity to increase WUE in water-poor environments. This might at least partly explain the alien-specific response observed in urban grasslands in Berlin, where overall, W_C_ was fairly low.

#### ^15^N enrichment factor

As in the case of iWUE_C_, no effect of species richness on Δδ^15^N_C_ was observed in the studied urban grasslands. This result contrasts with findings in central European semi-natural grasslands, where increasing plant diversity was associated to lower Δδ^15^N at the community and species level, indicating a more closed N cycle that was attributed to efficient N uptake via complementarity [57]. In our study, urban parameters were consistently identified as the main controlling factors for Δδ^15^N at the community, biogeographic status class and functional group level, which illustrates the predominant role of abiotic novelty over biodiversity or biotic novelty in regulating N cycling in urban grasslands in Berlin. Even though the relative importance of different urban parameters for different levels varied, all predictors were associated to an increasing trend in Δδ^15^N, that is, they favored a more open N cycle. Very likely, this common trend reflects the association of PopD, RailwD, FAR and impervious surface with urban N emissions. Increased anthropogenic N inputs stimulate N transformations (i.e. mineralization and nitrification), ultimately favoring N mobilization and loss in the form of leachable nitrate or nitrogenous gas [130], which might have led to a more N open cycle.

### Biodiversity-ecosystem functioning in urban grasslands

Our results on the positive relationship between total species richness and biomass illustrate the ability of urban grasslands to provide services in a comparable way to their non-urban counterparts. Previous studies have revealed the importance of urban grasslands for biodiversity conservation [24, 131] and cultural ecosystem services [132, 133]. Our findings highlight the relevance of biodiverse urban grasslands to ensure the provision of certain supporting and provisioning ecosystem services (i.e. primary production, carbon sequestration). In particular, we found that the positive relationship between species richness and AGB_C_ extensively described for European experimental grasslands [36] is maintained in the urban grasslands in Berlin even though they are intensively influenced by biotic and abiotic novelty (e.g. urbanization-related parameters) (Table 1). Our results agree with previous studies which reported a positive relationship ranging from linear to log linear with regression slopes that did not significantly differ from zero ([37] and references therein).

### Species level AGB, iWUE or Δδ^15^N

At the species level, we found no effect of species richness on photosynthetic performance, iWUE or Δδ^15^N, which suggests that the diversity of the surrounding vegetation did neither enhance nor hamper AGB production, water and N use of *C*. *epigejos* or *P*. *lanceolata* in the studied urban grasslands. Similar to our study, plant species richness did not affect ^13^C abundances in AGB of individual species in experimental grasslands, where photosynthetic assimilation seemed to be rather determined by the species position (i.e. height) in the plant canopy and the associated availability of light, air humidity and CO_2_ concentrations [134]. In accordance with the latter study, iWUE significantly increased with light availability (depicted by the SVF) in *C*. *epigejos* both in spring and summer, and at least in spring, this increase seemed to be driven by enhanced A and g_s_. The lack of SVF effects on *P*. *lanceolata* might be due to its comparably limited access to direct sunlight due to the lower position of this rosette species in the canopy. Height most likely also determined the contrasting seasonal variations observed in iWUE for the two studied species. iWUE_C.ep_ increased over the growing season, possibly due to increasing height and therefore greater access to light, which boosted A. However, in *P*. *lanceolata*, increasing height of the grassland canopy probably resulted in stronger shading of the rosette leaves, which increased g_s_ leading to a reduction in iWUE throughout the season. The significant decrease in F_v_/F_m_ and ΔF/F_m_′ in *P*. *lanceolat*a from spring to summer suggests a decline in photosynthetic capacity, in agreement with our above proposed explanation that the rate of AGB production decreased over the growing season due to increased shading. This is further supported by the results of the instant light-curves, which indicated an important decrease in intrinsic photosynthetic capacity (ETR_max_, ΔF/F_m_′ and ΔF/F_m_′ at PPDF_sat_) in *P*. *lanceolata* over the growing season but minimal seasonal variations in *C*. *epigejos*. The latter results, along with the lack of consistent effects of the degree of biotic novelty of the plots on photosynthetic parameters, iWUE or Δδ^15^N, suggest that abiotic novelty is the prevailing driver of photosynthetic performance and resource use efficiency of the selected individual species.

## Conclusions

While the biodiversity-ecosystem functioning relationship is critically understudied for urban grasslands, this study untangles a range of relationships between plant species richness, abiotic and biotic novelty and three important proxies of ecosystem functioning, namely aboveground biomass, intrinsic water use efficiency and ^15^N enrichment factor. Even though we identified a significant impact of abiotic novelty on AGB, iWUE and Δδ^15^N for various plant groups, the species richness-AGB_C_ relationship found in the studied urban grasslands in Berlin was comparable to that described in non-urban experimental grasslands in Europe. Our results support previous evidence that conserving and enhancing biodiversity in urban ecosystems is essential to warrant certain functions and ultimately their associated services (i.e. AGB production and primary production or carbon sequestration) and stress that management of urban grasslands in Berlin and other large cities should aim at preserving plant species richness. However, our results also suggest that preserving species richness is insufficient to ensure the provision of ecosystem services associated to other functions such as water and N use, which highlights the need for further studies addressing urban ecosystem functioning and exploring potential additional management measures directed to modify urban abiotic parameters.

## Supporting information

S1 Appendix Calculation of the Biotic Novelty Index (BNI). (DOCX)

S1 Table Presence (grey) and absence (white) of the two studied species in the selected grasslands in Berlin in 2017.
(DOCX)

S2 Table Overview of the random forests run for aboveground biomass (AGB), intrinsic water use efficiency (iWUE) and ^15^N enrichment factor (Δδ^15^N_C_) at the community, plant group (either biogeographic status, functional or combined) and species level in Berlin grasslands in 2017. (DOCX)

S3 Table Species present in the sampled quadrats in the grassland plots in Berlin in late summer 2017 along with the number and percentage of plots in which they were present. (DOCX)

S4 Table Geographic location of the grassland plots in Berlin in which *Berteroa incana* and *Conyza canadensis* were present in late summer 2017. (DOCX)

S1 File Partial dependence plots for the predictors selected by random forest models for aboveground biomass. (PDF)

S2 File Partial dependence plots for the predictors selected by random forest models for intrinsic water use efficiency. (PDF)

S3 File Partial dependence plots for the predictors selected by random forest models for N enrichment factor. (PDF)

S1 Dataset Digitized data extracted from Fig 3 in [36]. (XLSX)
