# Supplementary material for: Ecosystem functioning in urban grasslands: The role of biodiversity, plant invasions and urbanization
Source: PLoS One. 2019 Nov 22;14(11):e0225438. doi: 10.1371/journal.pone.0225438 (PMC6874358; doi:10.1371/journal.pone.0225438)
Supplement: S1 Appendix — (DOCX) [file pone.0225438.s001.docx]

**Calculation of the Biotic Novelty Index (BNI)**

There are six steps to calculating the BNI: (1) obtaining a trait matrix, (2) converting the trait matrix into a distance matrix, (3) obtaining the temporal coexistence matrix, (4) weighing the distance matrix by the temporal coexistence matrix, (5) multiplying the distance matrix by the species’ relative abundance and (6) calculating the sum of all pairwise comparisons from the distance matrix (1). Note that the calculation of the BNI corresponds to the calculation of Rao’s quadratic entropy (Rao 1982; Botta-Dukát 2005), but with a temporal coexistence component *c_ij_* added. In this way, the BNI is an additive partition of Rao’s quadratic entropy, quantifying only the functional diversity contributed by historically novel pairs of species in the community. The steps 1, 2, 5 and 6 are standard multivariate methods to obtain Rao’s index, steps 3 and 4 are the implementation of the temporal coexistence component. Information on how to calculate Rao’s quadratic entropy can be found elsewhere (see Rao 1964 and Botta-Dukát 2005 for example). Here, we describe how the temporal coexistence component was calculated.

**The temporal coexistence component**

The pairwise distance matrix of Rao’s quadratic entropy becomes weighted by the pairwise temporal coexistence component, which is also in matrix form. To obtain the matrix, it needs to be specified for each species whether it belongs to the historical native species pool or whether the species is alien. We used information of species’ first records (and/or time of establishment) from the BiolFlor database (Klotz 2002) and a local expert (Birgit Seitz, Technical University Berlin) to specify the time when an alien species arrived in the Berlin area. From this information, the residence time for each species was calculated. The residence time tells us how many years before today each species was introduced or has been established. For example, a species that was introduced in 1719 has a residence time of 300 years in the year 2019 (the current year). Next, in a normalization step the resident times were brought into the range [0,1] expressed by:

$$r_{i}'=1-\frac{r_{i}-r_{min}}{r_{max}-r_{min}}$$

where *r_i_*’ is the normalized residence time of species *i*, *r_i_* is the residence time of species *i*, *r_min_* is the minimum residence time of all species and *r_max_* the maximum residence time of all species. Once the normalized residence time was calculated for each species, for each pair of species the temporal coexistence coefficient could be calculated as follows:

$$c_{ij}=max(r_{i}^{'},r_{j}^{'})$$

where *c_ij_* is the temporal coexistence coefficient of species *i* and *j*, *r_i_*’ is the normalized residence time of species *i* and *r_j_*’ is the normalized residence time of species *j.* From these pairwise temporal coexistence coefficients a matrix was obtained that functions as a weighting factor for the pairwise distance matrix.

**1**. Schittko C, Bernard-Verdier M, Heger T, Buchholz S, Kowarik I, von der Lippe M, Seitz B, Joshi J, Jeschke JM. A multidimensional framework for measuring biotic novelty: How novel is a community? bioRxiv. 2019:824045. Cited 31 October 2019.
