# Supplementary material for: Ecosystem functioning in urban grasslands: The role of biodiversity, plant invasions and urbanization
Source: PLoS One. 2019 Nov 22;14(11):e0225438. doi: 10.1371/journal.pone.0225438 (PMC6874358; doi:10.1371/journal.pone.0225438)
Supplement: S1 Table — Presence (grey) and absence (white) of the two studied species in the selected grasslands in Berlin in 2017. (DOCX) [file pone.0225438.s002.docx]

**S1 Table. Presence (grey) and absence (white) of the two studied species in the selected grasslands in Berlin in 2017.**

|  |  |  |  |  |  |  |
| --- | --- | --- | --- | --- | --- | --- |
|  | Plot | *C. epigejos* | | *P. lanceolata* | |  |
|  |  | Spring | Summer | Spring | Summer |  |
|  | Nh_04 |  |  |  |  |  |
|  | Nh_05 |  |  |  |  |  |
|  | Nh_10 |  |  |  |  |  |
|  | Nh_201 |  |  |  |  |  |
|  | Nh_51 |  |  |  |  |  |
|  | NL_09 |  |  |  |  |  |
|  | Nl_14 |  |  |  |  |  |
|  | Nl_200 |  |  |  |  |  |
|  | Nl_206 |  |  |  |  |  |
|  | Nl_208 |  |  |  |  |  |
|  | Nl_220 |  |  |  |  |  |
|  | Nm_09 |  |  |  |  |  |
|  | Nm_14 |  |  |  |  |  |
|  | Nm_201 |  |  |  |  |  |
|  | Oh_01 |  |  |  |  |  |
|  | Oh_02 |  |  |  |  |  |
|  | Oh_03 |  |  |  |  |  |
|  | Oh_04 |  |  |  |  |  |
|  | Ol_55 |  |  |  |  |  |
|  | Om_05 |  |  |  |  |  |
|  | n | 13 | 17 | 9 | 12 |  |
|  |  |  |  |  |  |  |
