# Supplementary material for: Ecosystem functioning in urban grasslands: The role of biodiversity, plant invasions and urbanization
Source: PLoS One. 2019 Nov 22;14(11):e0225438. doi: 10.1371/journal.pone.0225438 (PMC6874358; doi:10.1371/journal.pone.0225438)
Supplement: S2 Table — (DOCX) [file pone.0225438.s003.docx]

**S2 Table. Overview of the random forests run for aboveground biomass (AGB), intrinsic water use efficiency (iWUE) and ^15^N enrichment factor (Δδ^15^N_C_) at the community, plant group (either geographic status, functional or combined) and species level in Berlin grasslands in 2017. Grey and white boxes indicate computed and not computed models, respectively.**

| **Level** | **AGB** | **iWUE** | **Δδ^15^N** |
| --- | --- | --- | --- |
| Community |  |  |  |
| Aliens |  |  |  |
| Natives |  |  |  |
| Graminoids |  |  |  |
| Forbs |  |  |  |
| Legumes |  |  |  |
| Native graminoids |  |  |  |
| Alien graminoids |  |  |  |
| Native forbs |  |  |  |
| Alien forbs |  |  |  |
| Native legumes |  |  |  |
| *C.epigejos* |  |  |  |
| *P.lanceolata* |  |  |  |
