# Supplementary material for: Ecosystem functioning in urban grasslands: The role of biodiversity, plant invasions and urbanization
Source: PLoS One. 2019 Nov 22;14(11):e0225438. doi: 10.1371/journal.pone.0225438 (PMC6874358; doi:10.1371/journal.pone.0225438)
Supplement: S3 Table — (DOCX) [file pone.0225438.s004.docx]

**S3 Table. Species present in the sampled quadrats in the grassland plots in Berlin in late summer 2017 along with the number and percentage of plots in which they were present.** These data refer to 17 grassland plots, as in 3 plots the data was not recorded up to the species level, but up to the functional and biogeographical origin level instead.
