# Supplementary material for: Ecosystem functioning in urban grasslands: The role of biodiversity, plant invasions and urbanization
Source: PLoS One. 2019 Nov 22;14(11):e0225438. doi: 10.1371/journal.pone.0225438 (PMC6874358; doi:10.1371/journal.pone.0225438)
Supplement: S4 Table — (DOCX) [file pone.0225438.s005.docx]

**S4 Table. Geographic location of the grassland plots in Berlin in which *Berteroa incana* and *Conyza canadensis* were present (grey) or absent (white) in late summer 2017.**

|  |  |  |  |  |  |  |  |
| --- | --- | --- | --- | --- | --- | --- | --- |
|  | Plot | Geographic location | | *Berteroa*  *incana* | *Conyza*  *canadensis* | |  |
|  |  | Longitude | Latitude |  |  |  |  |
|  | Nh_04 | 13.30888 | 52.40597 |  |  | |  |
|  | Nh_05 | 13.40603 | 52.47541 |  |  | |  |
|  | Nh_10 | 13.51432 | 52.44011 |  |  | |  |
|  | Nh_201 | 13.54843 | 52.48448 |  |  | |  |
|  | Nh_51 | 13.24265 | 52.4882 |  |  | |  |
|  | NL_09 | 13.37276 | 52.48854 |  |  | |  |
|  | Nl_14 | 13.38538 | 52.53438 |  |  | |  |
|  | Nl_200 | 13.37698 | 52.53825 |  |  | |  |
|  | Nl_206 | 13.3465 | 52.56343 |  |  | |  |
|  | Nl_208 | 13.55584 | 52.43955 |  |  | |  |
|  | Nl_220 | 13.35497 | 52.47755 |  |  | |  |
|  | Nm_09 | 13.35908 | 52.46165 |  |  | |  |
|  | Nm_14 | 13.52209 | 52.4688 |  |  | |  |
|  | Nm_201 | 13.28078 | 52.50321 |  |  | |  |
|  | Oh_01 | 13.58918 | 52.47647 |  |  | |  |
|  | Oh_02 | 13.57169 | 52.50037 |  |  | |  |
|  | Oh_03 | 13.20594 | 52.44694 |  |  |  |  |
|  | Oh_04 | 13.20171 | 52.58678 |  |  | |  |
|  | Ol_55 | 13.18328 | 52.41555 |  |  | |  |
|  | Om_05 | 13.24726 | 52.58265 |  |  | |  |
|  |  |  |  |  |  |  |  |
