# Supplementary material for: Ecosystem functioning in urban grasslands: The role of biodiversity, plant invasions and urbanization
Source: PLoS One. 2019 Nov 22;14(11):e0225438. doi: 10.1371/journal.pone.0225438 (PMC6874358; doi:10.1371/journal.pone.0225438)
Supplement: S1 File — (PDF) [file pone.0225438.s006.pdf]

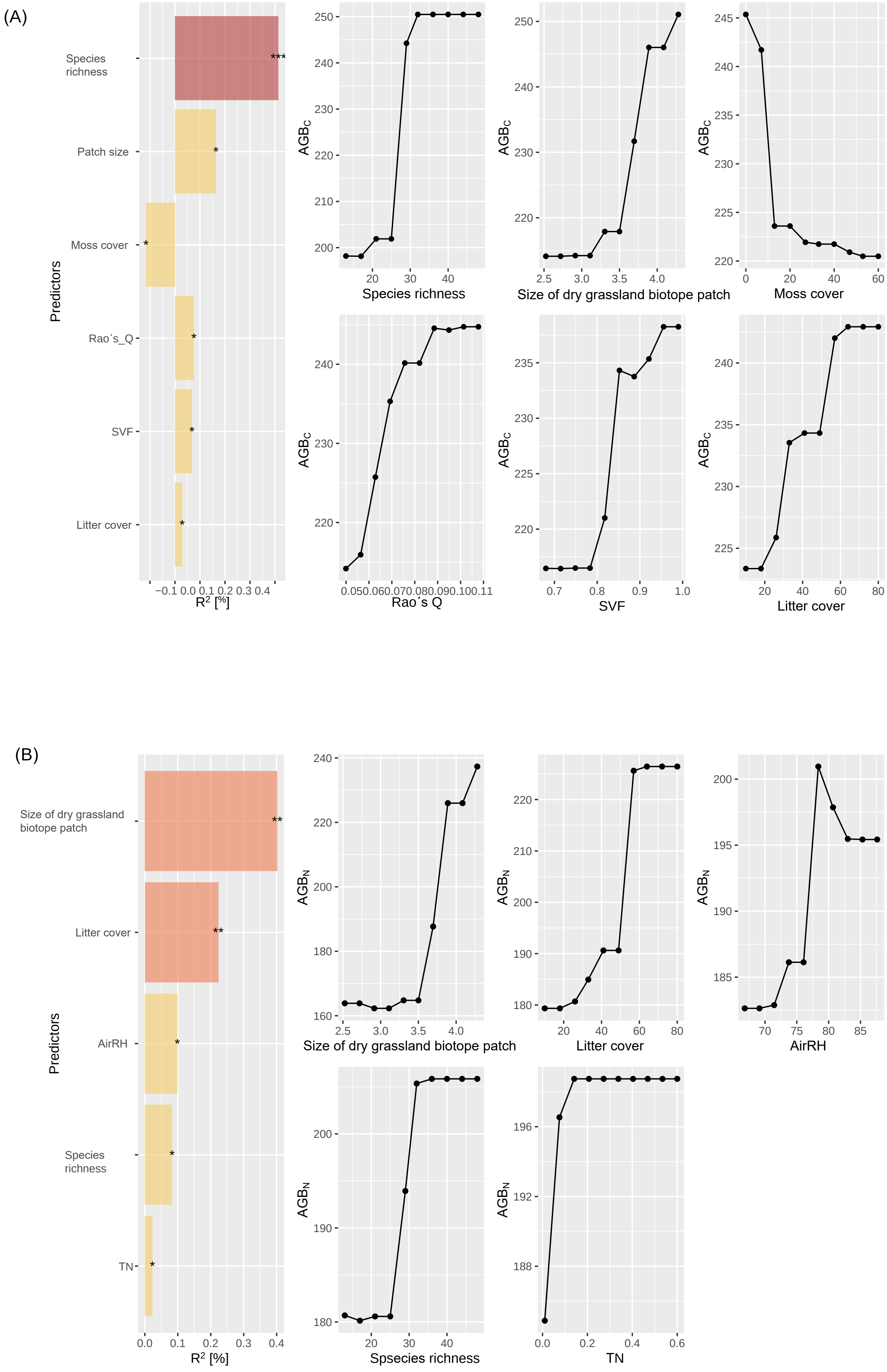

(A)
Species richness
Patch size
Moss cover
Rao´s_Q
SVF
Litter cover
Predictors
R² [%]
AGB_C
Species richness
Size of dry grassland biotope patch
Moss cover
Rao´s Q
SVF
Litter cover
(B)
Size of dry grassland biotope patch
Litter cover
AirRH
Species richness
TN
Predictors
R² [%]
AGB_N
Size of dry grassland biotope patch
Litter cover
AirRH
Spsecies richness
TN

(C)

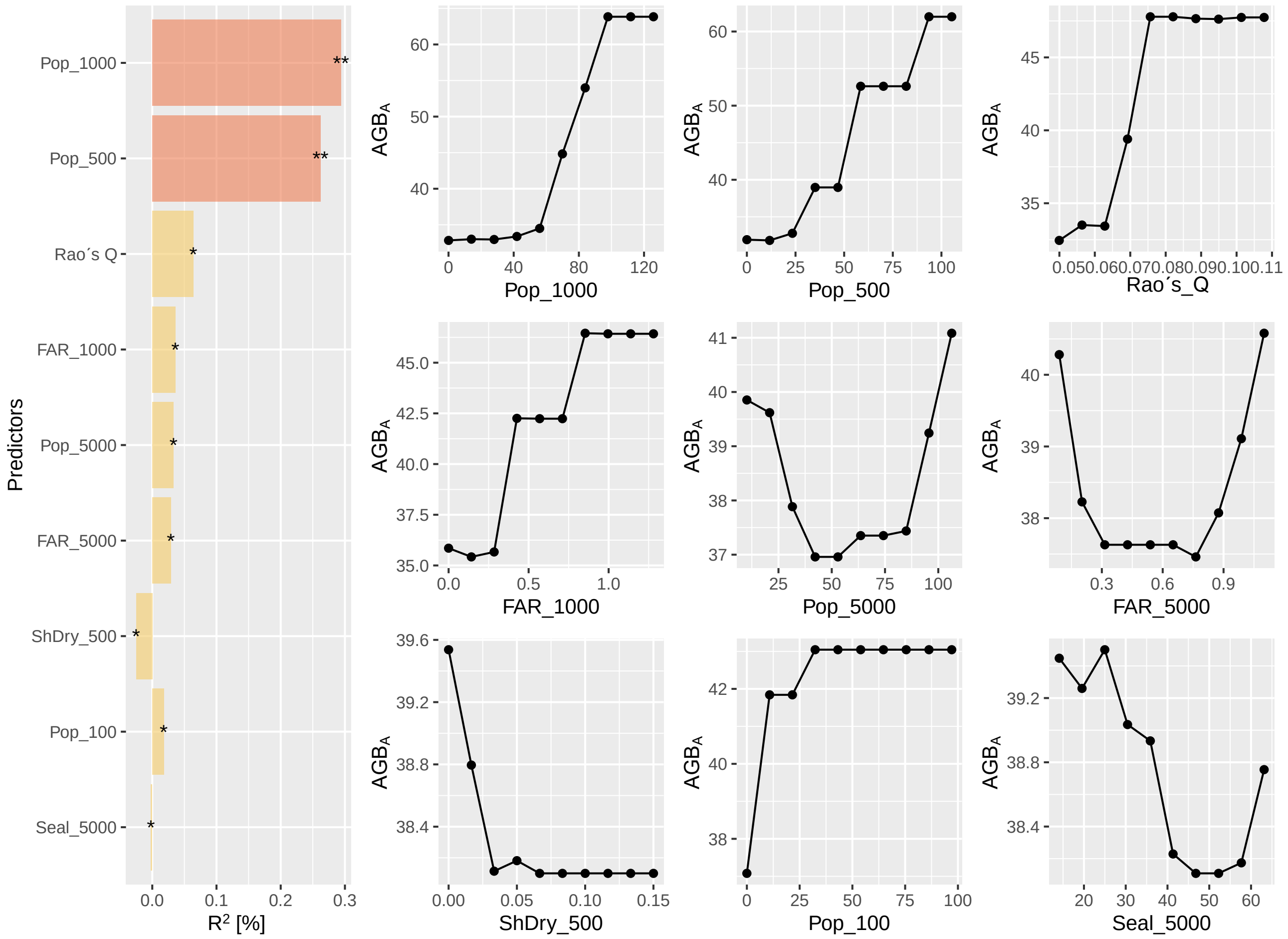
Predictors
Pop_1000
Pop_500
Rao´s Q
FAR_1000
Pop_5000
FAR_5000
ShDry_500
Pop_100
Seal_5000
**
**
*
R² [%]
AGB_A
Pop_1000
Pop_500
Rao´s_Q
FAR_1000
Pop_5000
FAR_5000
ShDry_500
Pop_100
Seal_5000

(D)

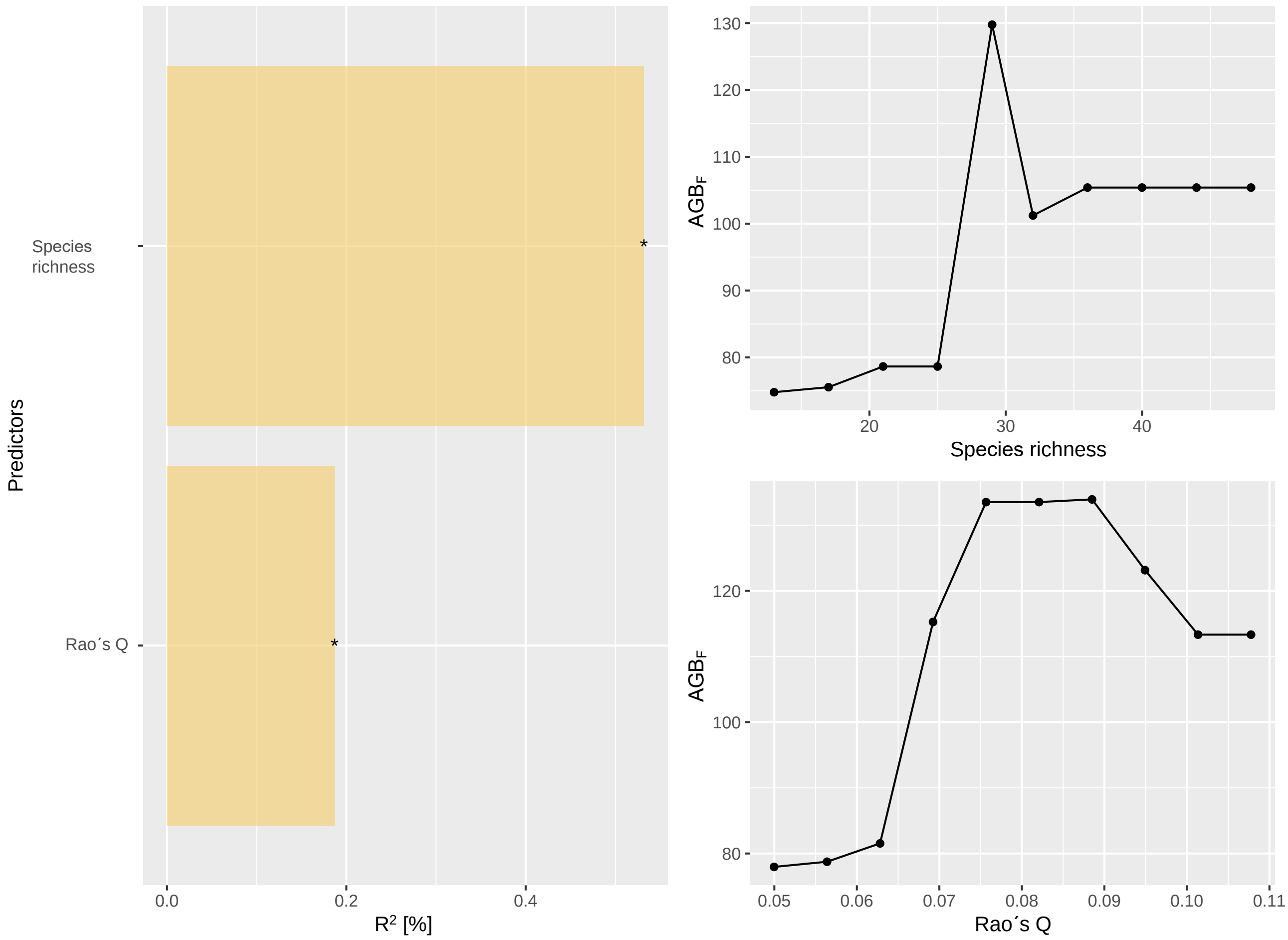
Predictors
Species richness
Rao´s Q
*
R² [%]
AGB_F
Species richness
Rao´s Q

(E)

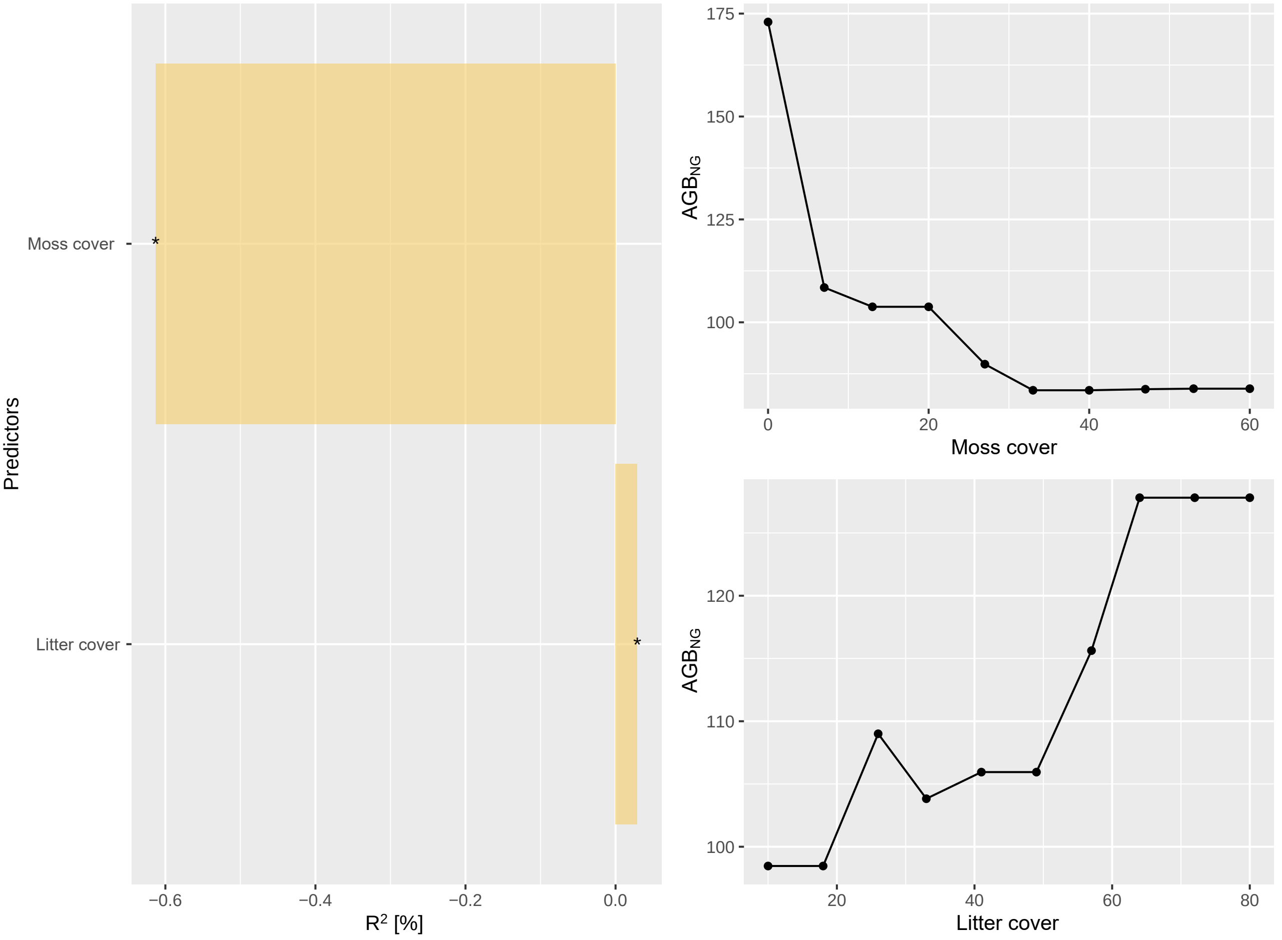
Moss cover
Litter cover
Predictors
−0.6
−0.4
−0.2
0.0
R² [%]
175
150
125
100
AGB_NG
0
20
40
60
Moss cover
120
110
100
AGB_NG
20
40
60
80
Litter cover

(F)

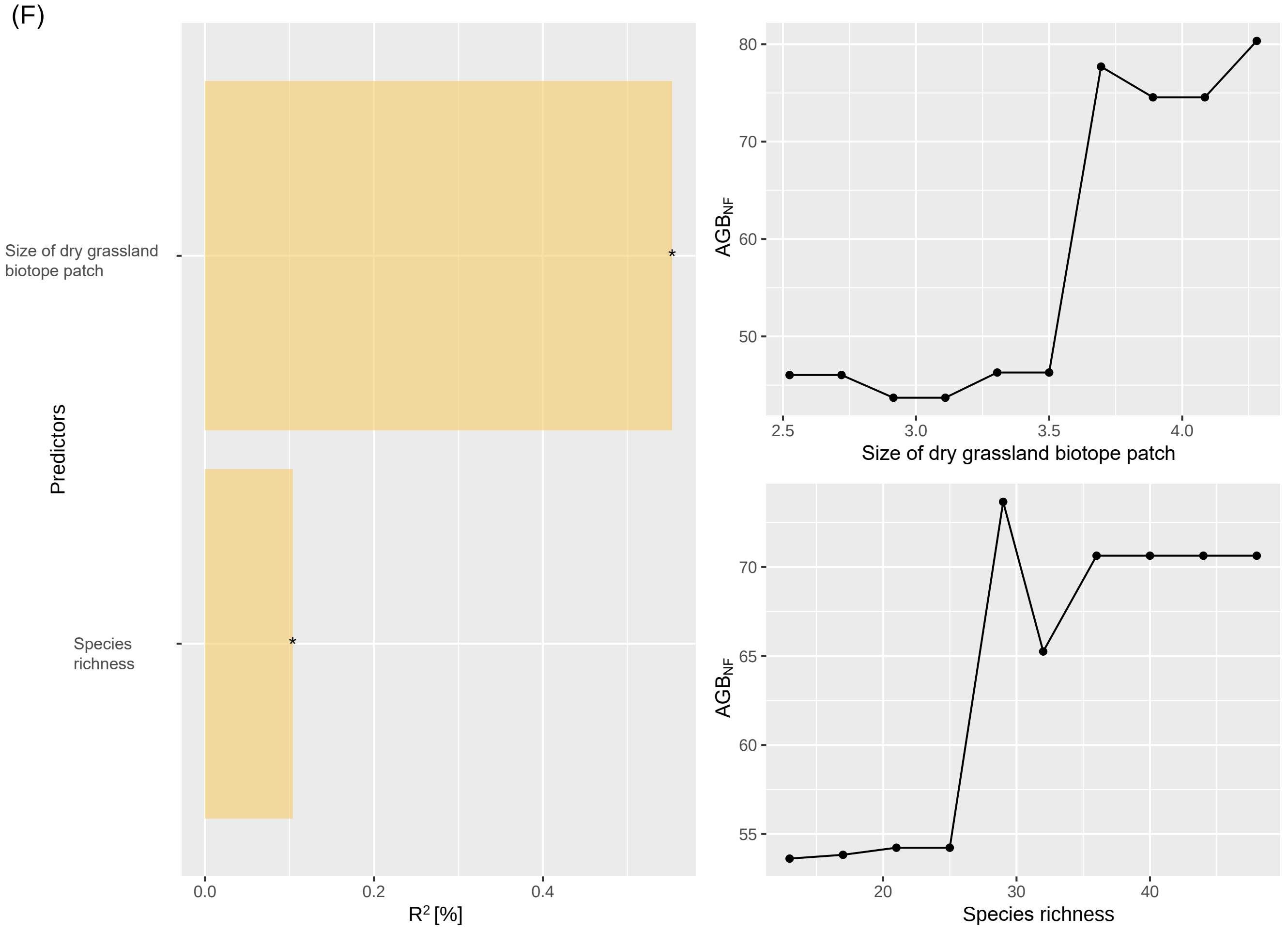
Size of dry grassland biotope patch
Species richness
Predictors
0.0
0.2
0.4
R² [%]
80
70
60
50
AGB_NF
2.5
3.0
3.5
4.0
Size of dry grassland biotope patch
70
65
60
55
AGB_NF
20
30
40
Species richness

(G)

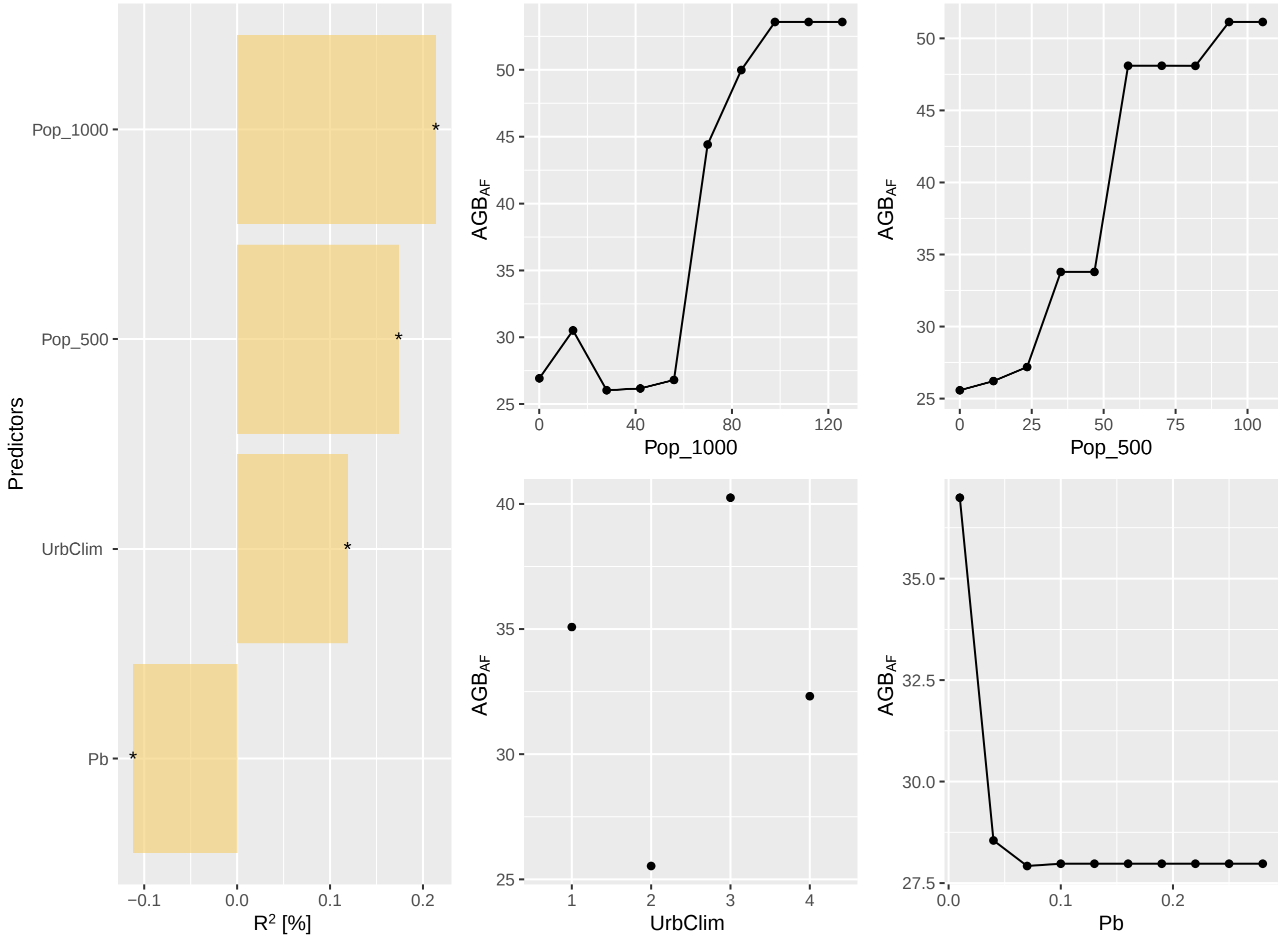

Pop_1000
Pop_500
UrbClim
Pb
Predictors
R² [%]
−0.1
0.0
0.1
0.2
AGB_AF
Pop_1000
Pop_500
UrbClim
Pb

S5 File. Partial dependence plots for the predictors selected by random forest models for aboveground biomass (AGB) of (A) the whole plant community (AGB_C), (B) natives (AGB_N), (C) aliens (AGB_A), (D) forbs (AGB_F), (E) native graminoids (AGB_{NG}), (F) native forbs (AGB_{NF}) and (G) alien forbs (AGB_{AF}). Note that a partial dependency plot is used not to confirm the effect size but the association pattern including effect direction, since due to normalization, the ranges of y-axes do not directly correspond to the range of the variable. The left panels indicate the relative importance of the explanatory variables selected for each random forest model. The significance of each predictor selected for the models is indicated by asterisks: * $p<0.05$, ** $p<0.01$ and *** $p<0.001$).
