# Supplementary material for: Ecosystem functioning in urban grasslands: The role of biodiversity, plant invasions and urbanization
Source: PLoS One. 2019 Nov 22;14(11):e0225438. doi: 10.1371/journal.pone.0225438 (PMC6874358; doi:10.1371/journal.pone.0225438)
Supplement: S2 File — (PDF) [file pone.0225438.s007.pdf]

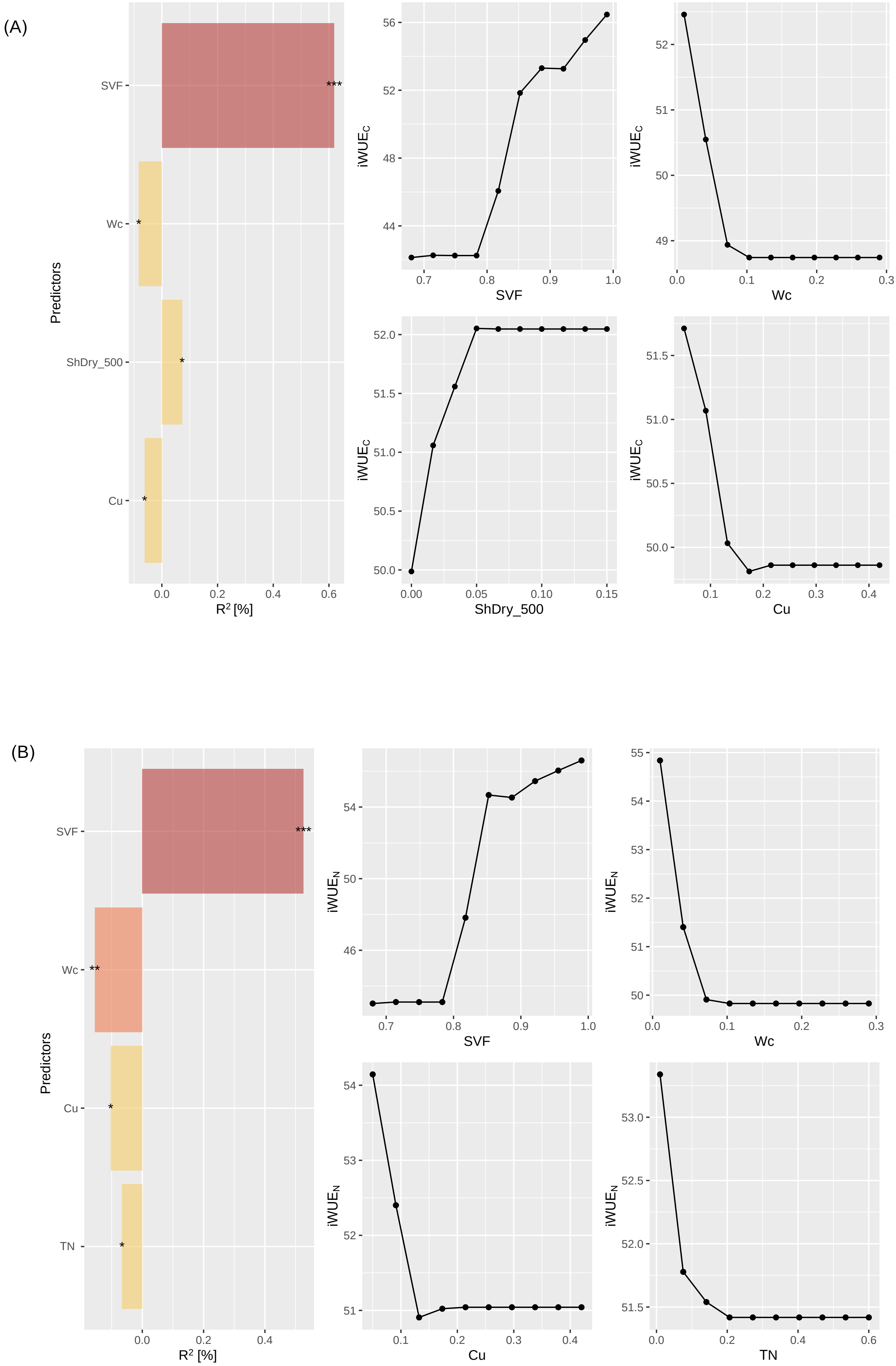

(A)
Predictors
SVF
Wc
ShDry_500
Cu
***
*
*
*
R^2 [%]
0.0
0.2
0.4
0.6
iWUE$_C$
SVF
Wc
ShDry_500
Cu
(B)
Predictors
SVF
Wc
Cu
TN
***
**
*
*
R^2 [%]
iWUE$_N$
SVF
Wc
Cu
TN

(C)

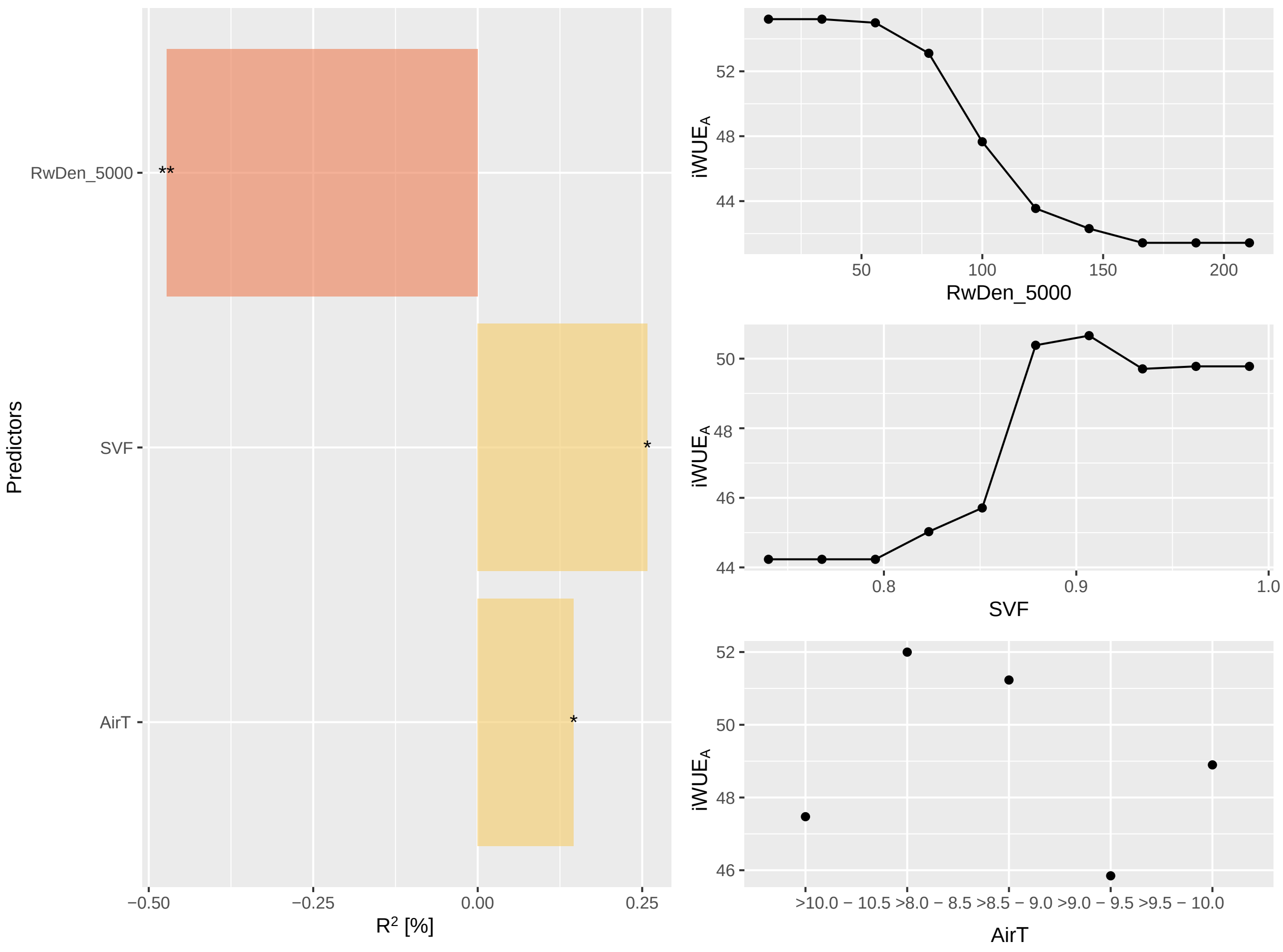
Predictors
RwDen_5000
**
SVF
*
AirT
*
−0.50
−0.25
0.00
0.25
R² [%]
iWUEA
52
48
44
50
100
150
200
RwDen_5000
50
48
46
44
0.8
0.9
1.0
SVF
52
50
48
46
>10.0 − 10.5 >8.0 − 8.5 >8.5 − 9.0 >9.0 − 9.5 >9.5 − 10.0
AirT

(D)

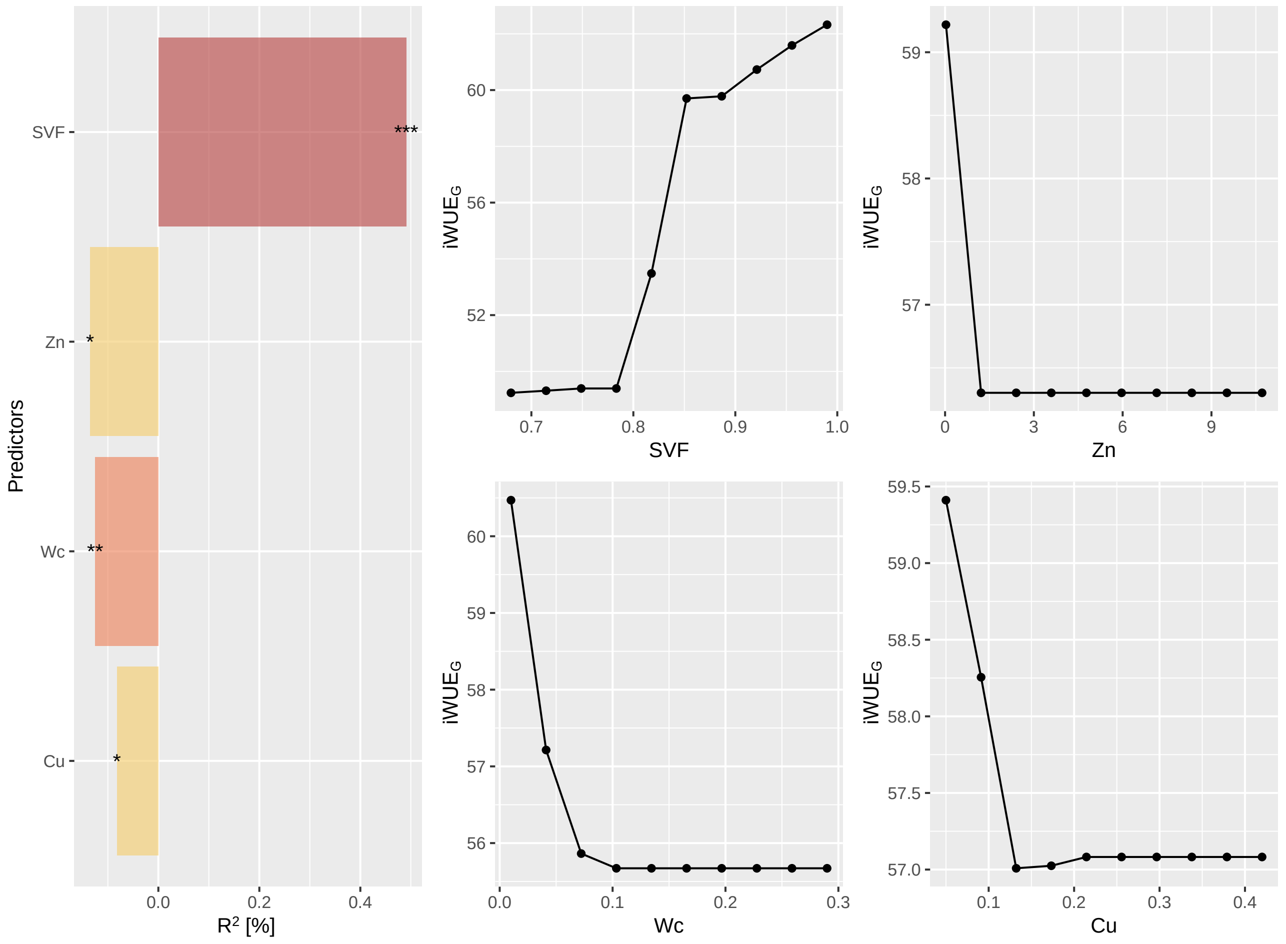
Predictors
SVF
***
Zn
*
Wc
**
Cu
*
0.0
0.2
0.4
R² [%]
iWUEG
60
56
52
0.7
0.8
0.9
1.0
SVF
59
58
57
0
3
6
9
Zn
60
59
58
57
56
0.0
0.1
0.2
0.3
Wc
59.5
59.0
58.5
58.0
57.5
57.0
0.1
0.2
0.3
0.4
Cu

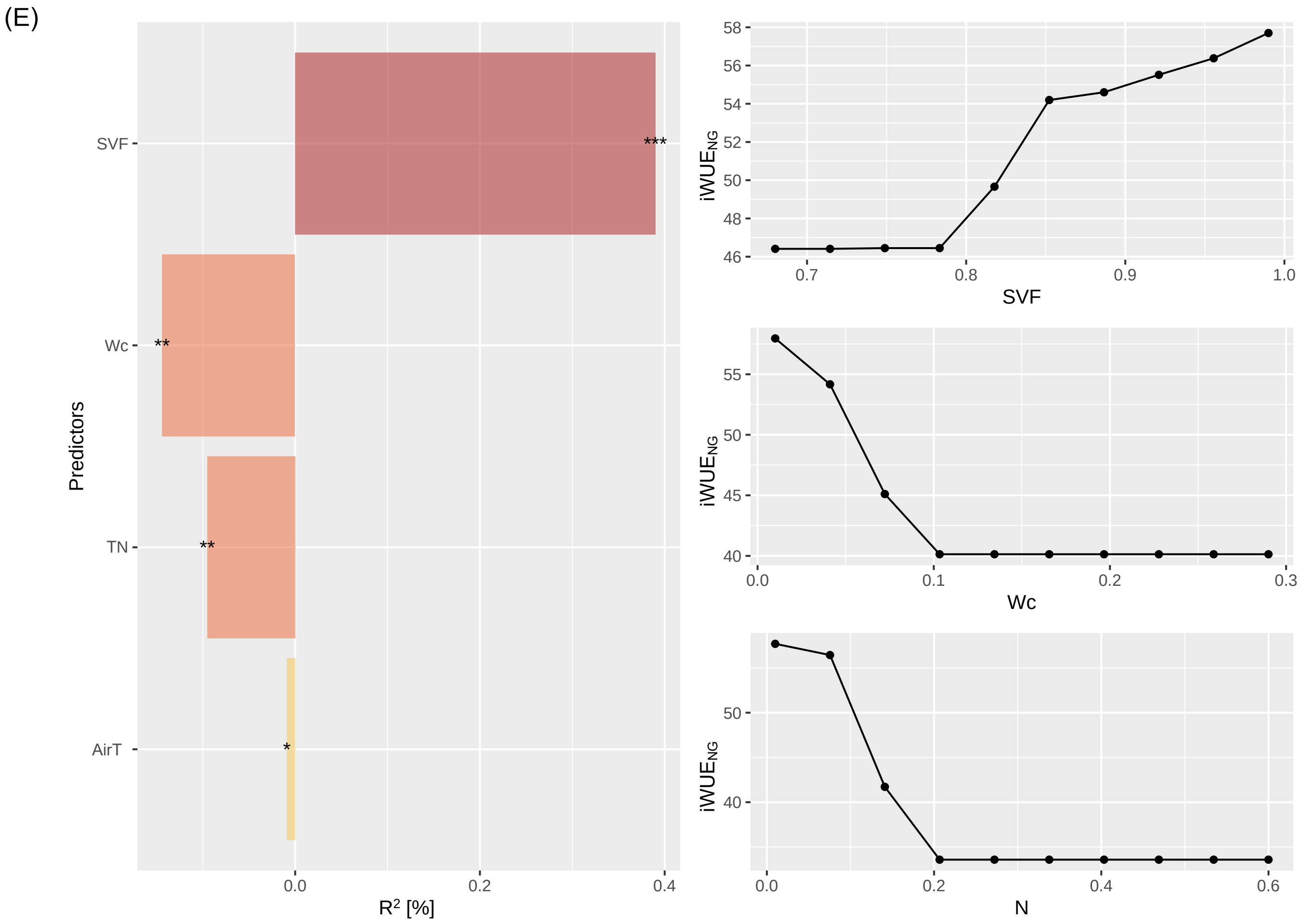

(E)
SVF
Wc
TN
AirT
Predictors
***
**
**
*
0.0
0.2
0.4
R² [%]
iWUE_NG
SVF
Wc
N

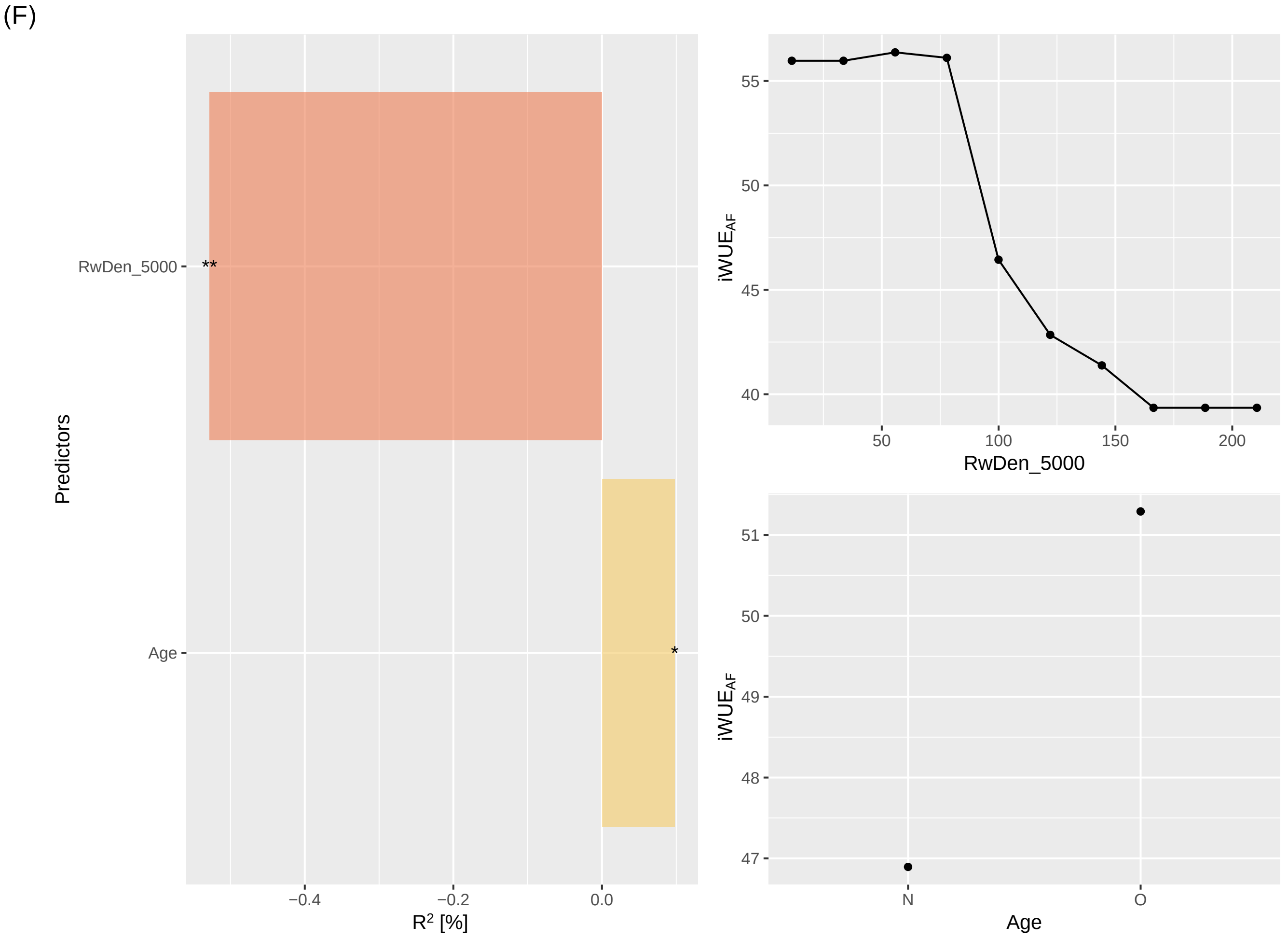

(F)
RwDen_5000
Age
Predictors
**
*
−0.4
−0.2
0.0
R² [%]
iWUE_AF
RwDen_5000
Age
N
O

S6 File. Partial dependence plots for the predictors selected by random forest models for intrinsic water use efficiency (iWUE) of (A) the whole plant community ($iWUE_C$), (B) natives (iWUEN), (C) aliens ($iWUE_A$), (D) graminoids ($iWUE_G$), (E) native graminoids ($iWUE_{NG}$) and (F) alien forbs ($iWUE_{AF}$). Note that a partial dependency plot is used not to confirm the effect size but the association patternincluding effect direction, since due to normalization, the ranges of y-axes do not directly correspond to the range of the variable. The left panels indicate the relative importance of the explanatory variables selected for each random forest model. The significance of each predictor selected for the models is indicated by asterisks: * $p<0.05$, ** $p<0.01$ and *** $p<0.001$).
