# Supplementary material for: Ecosystem functioning in urban grasslands: The role of biodiversity, plant invasions and urbanization
Source: PLoS One. 2019 Nov 22;14(11):e0225438. doi: 10.1371/journal.pone.0225438 (PMC6874358; doi:10.1371/journal.pone.0225438)
Supplement: S3 File — (PDF) [file pone.0225438.s008.pdf]

(A)

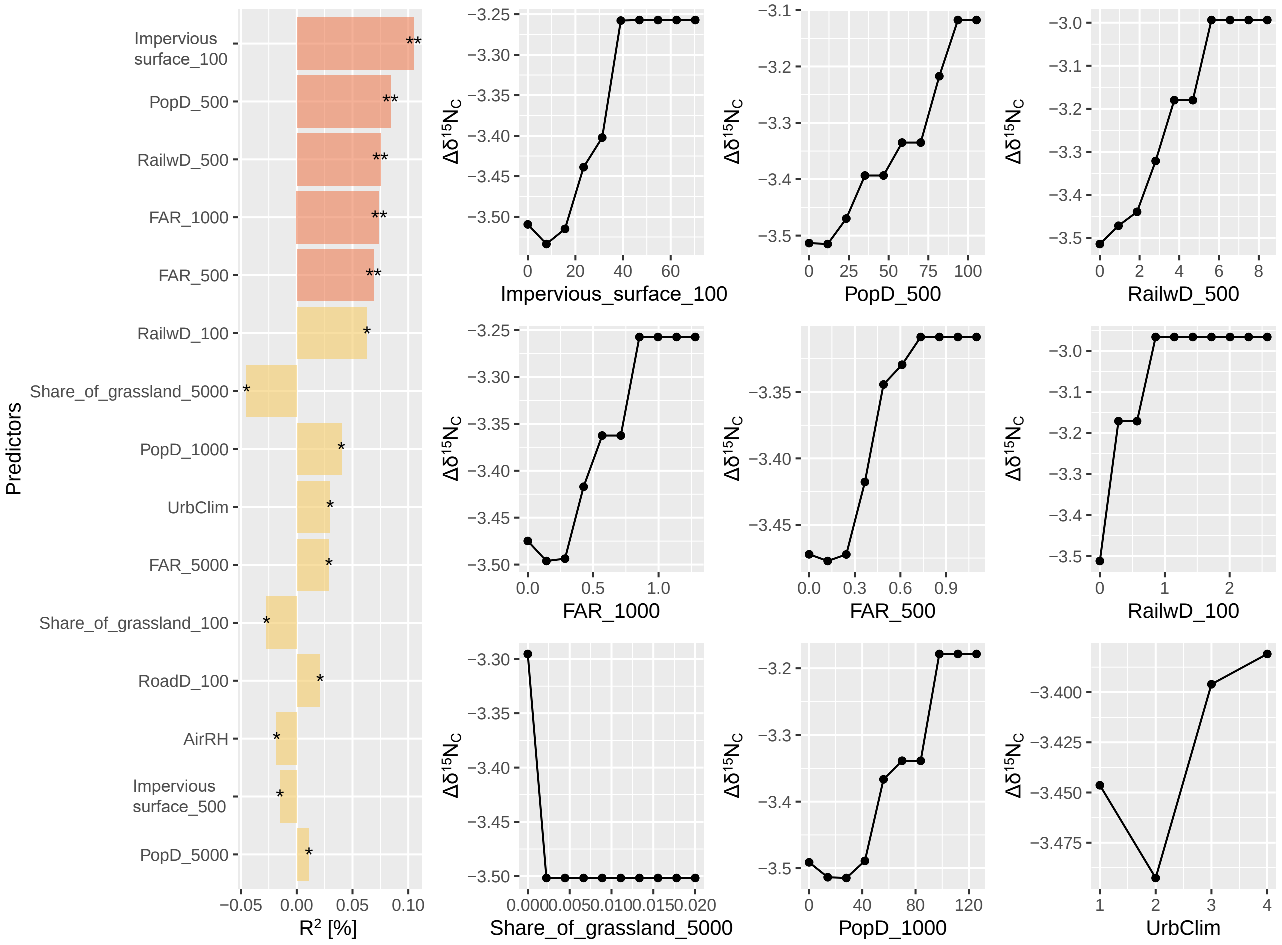

Predictors
Impervious surface_100
PopD_500
RailwD_500
FAR_1000
FAR_500
RailwD_100
Share_of_grassland_5000
PopD_1000
UrbClim
FAR_5000
Share_of_grassland_100
RoadD_100
AirRH
Impervious surface_500
PopD_5000
R² [%]
Δδ15NC
Impervious_surface_100
PopD_500
RailwD_500
FAR_1000
FAR_500
RailwD_100
Share_of_grassland_5000
PopD_1000
UrbClim

(B)

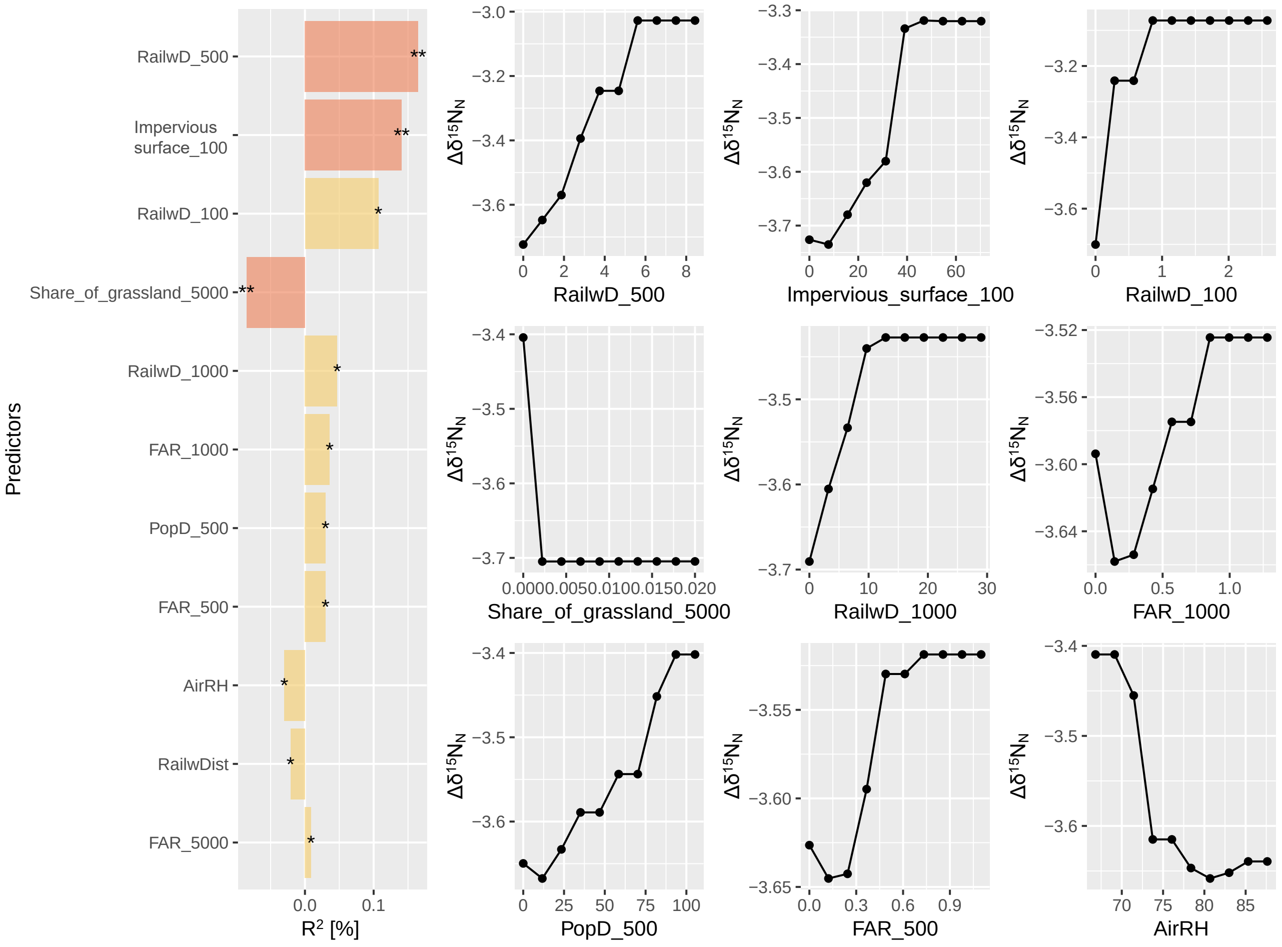

Predictors
RailwD_500
Impervious surface_100
RailwD_100
Share_of_grassland_5000
RailwD_1000
FAR_1000
PopD_500
FAR_500
AirRH
RailwDist
FAR_5000
R² [%]
Δδ15NN
RailwD_500
Impervious_surface_100
RailwD_100
Share_of_grassland_5000
RailwD_1000
FAR_1000
PopD_500
FAR_500
AirRH

(C)

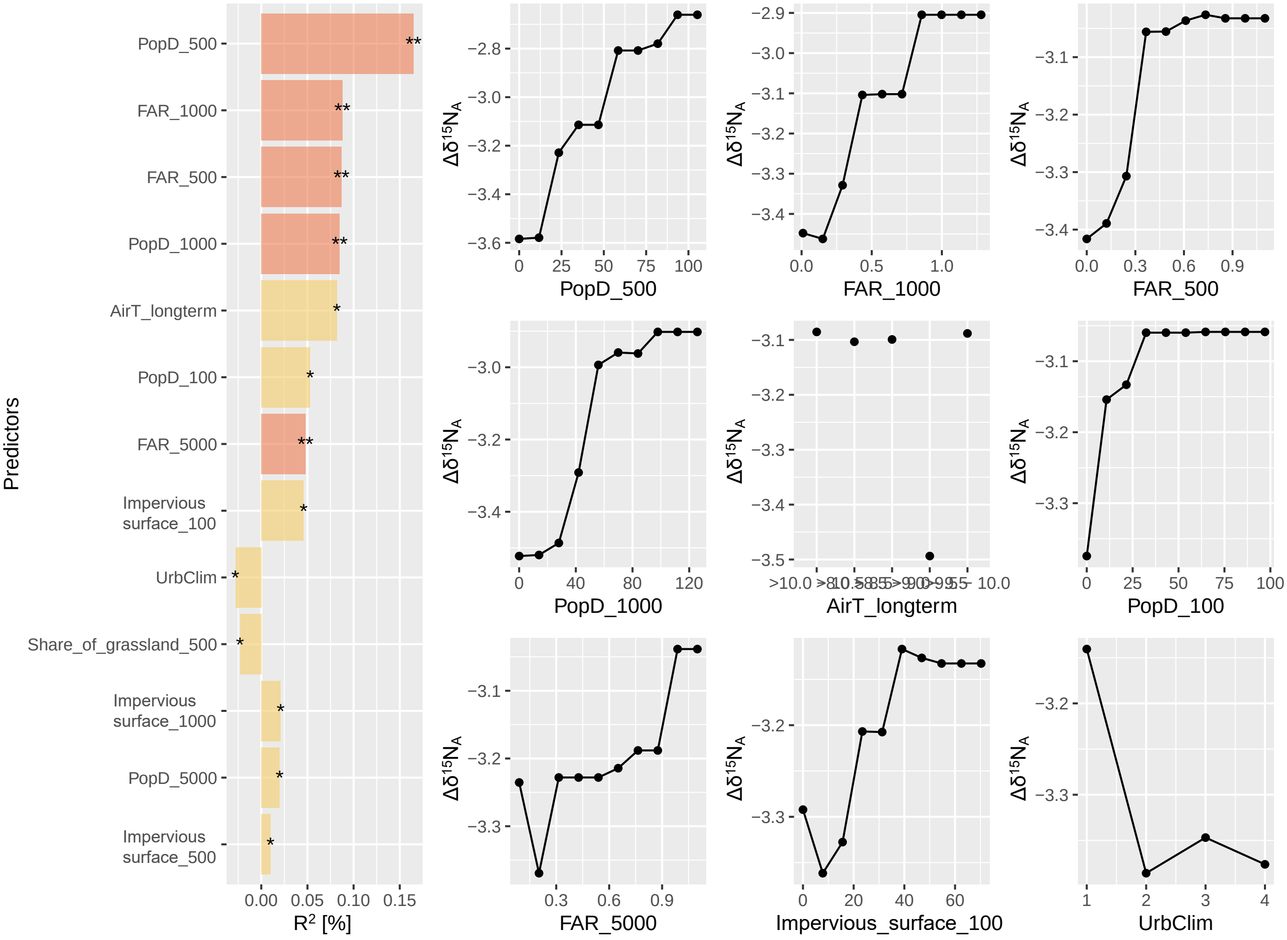
Predictors
PopD_500
FAR_1000
FAR_500
PopD_1000
AirT_longterm
PopD_100
FAR_5000
Impervious surface_100
UrbClim
Share_of_grassland_500
Impervious surface_1000
PopD_5000
Impervious surface_500
R² [%]
0.00 0.05 0.10 0.15
Δδ15NA
PopD_500
FAR_1000
FAR_500
PopD_1000
AirT_longterm
PopD_100
FAR_5000
Impervious_surface_100
UrbClim

(D)

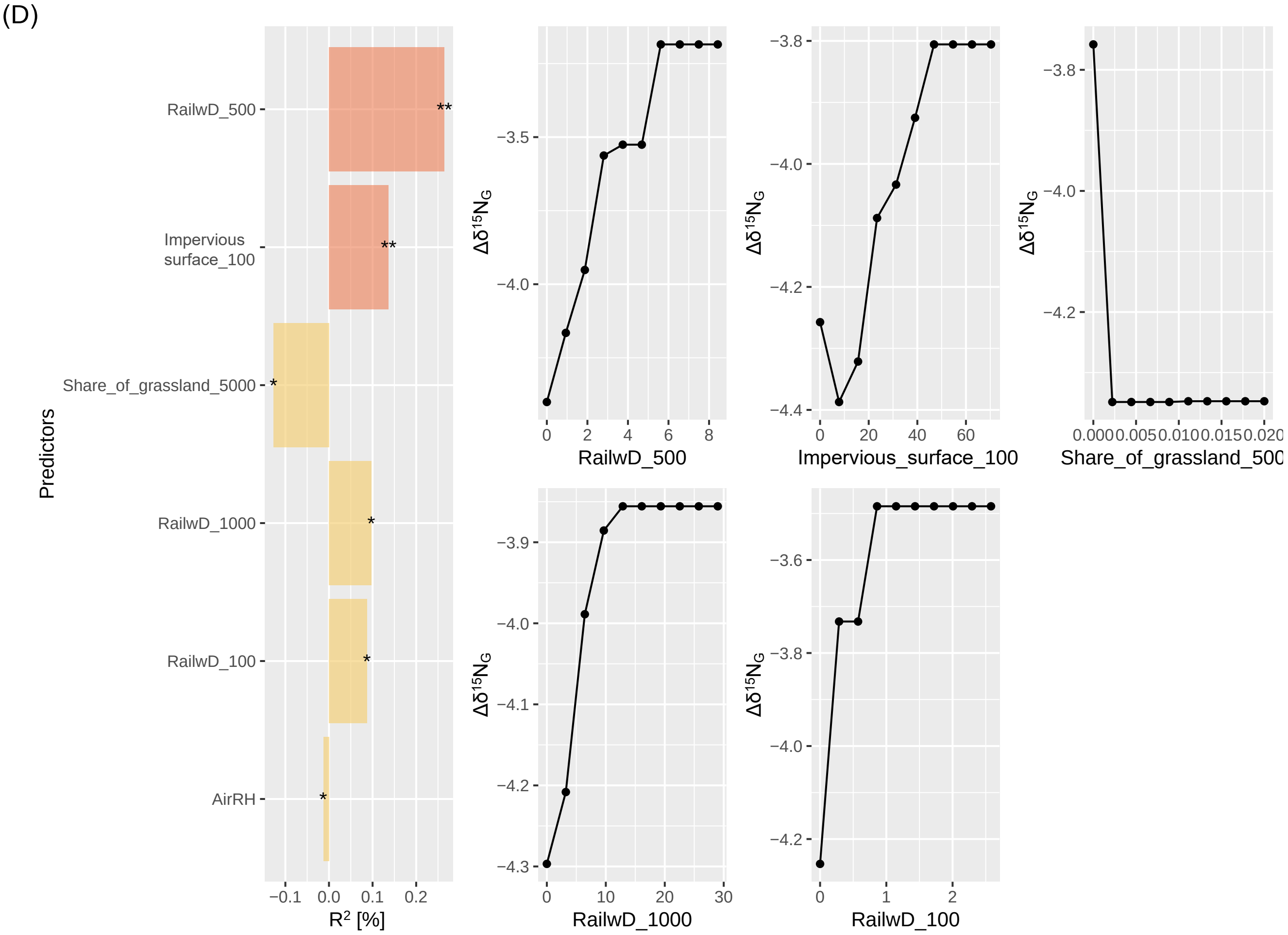
Predictors
RailwD_500
Impervious surface_100
Share_of_grassland_5000
RailwD_1000
RailwD_100
AirRH
R² [%]
−0.1 0.0 0.1 0.2
Δδ15NG
RailwD_500
Impervious_surface_100
Share_of_grassland_500
RailwD_1000
RailwD_100

(E)

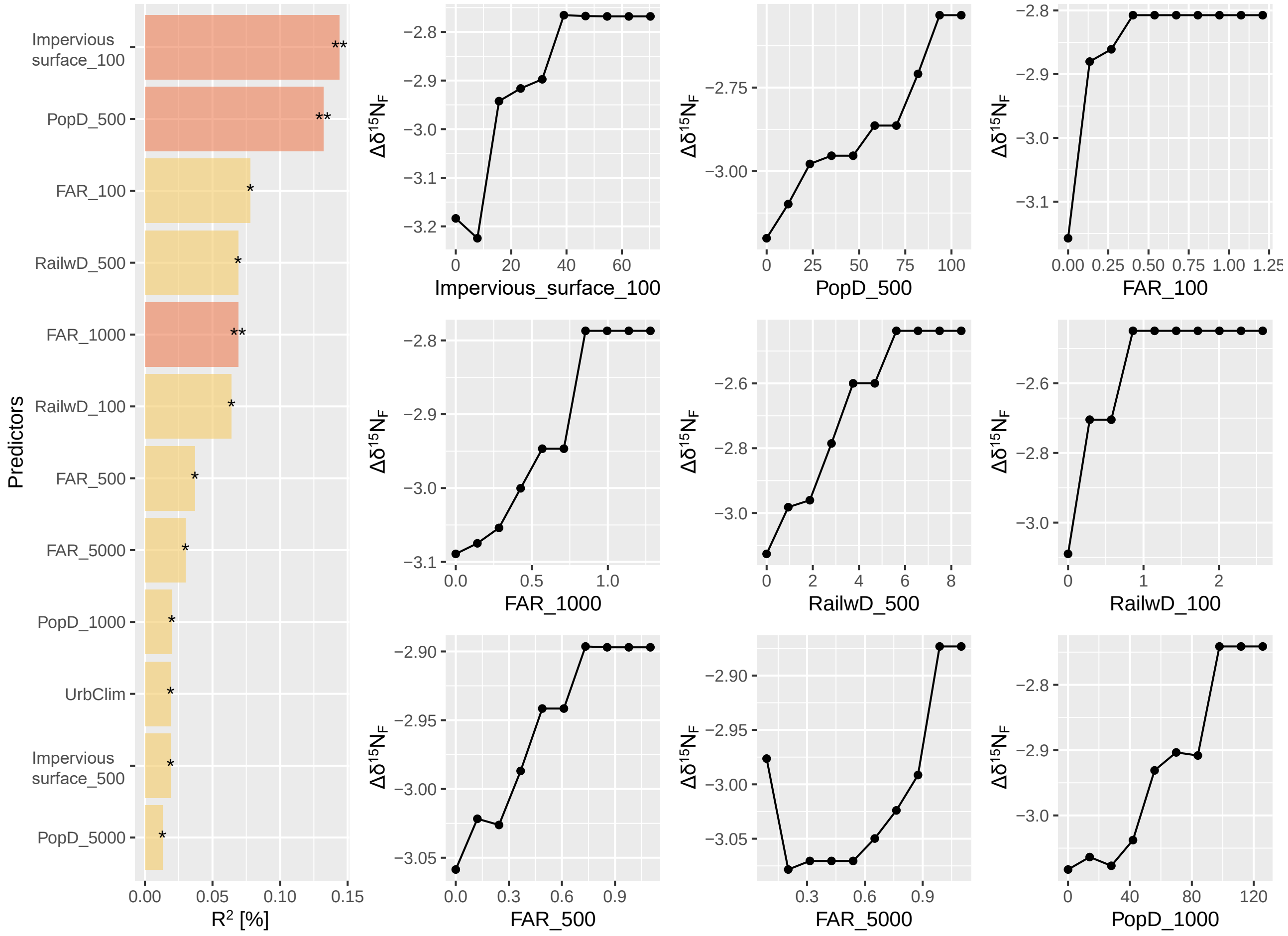
Predictors
Impervious surface_100
PopD_500
FAR_100
RailwD_500
FAR_1000
RailwD_100
FAR_500
FAR_5000
PopD_1000
UrbClim
Impervious surface_500
PopD_5000
R² [%]
Δδ15NF
Impervious_surface_100
PopD_500
FAR_100
FAR_1000
RailwD_500
RailwD_100
FAR_500
FAR_5000
PopD_1000

(F)

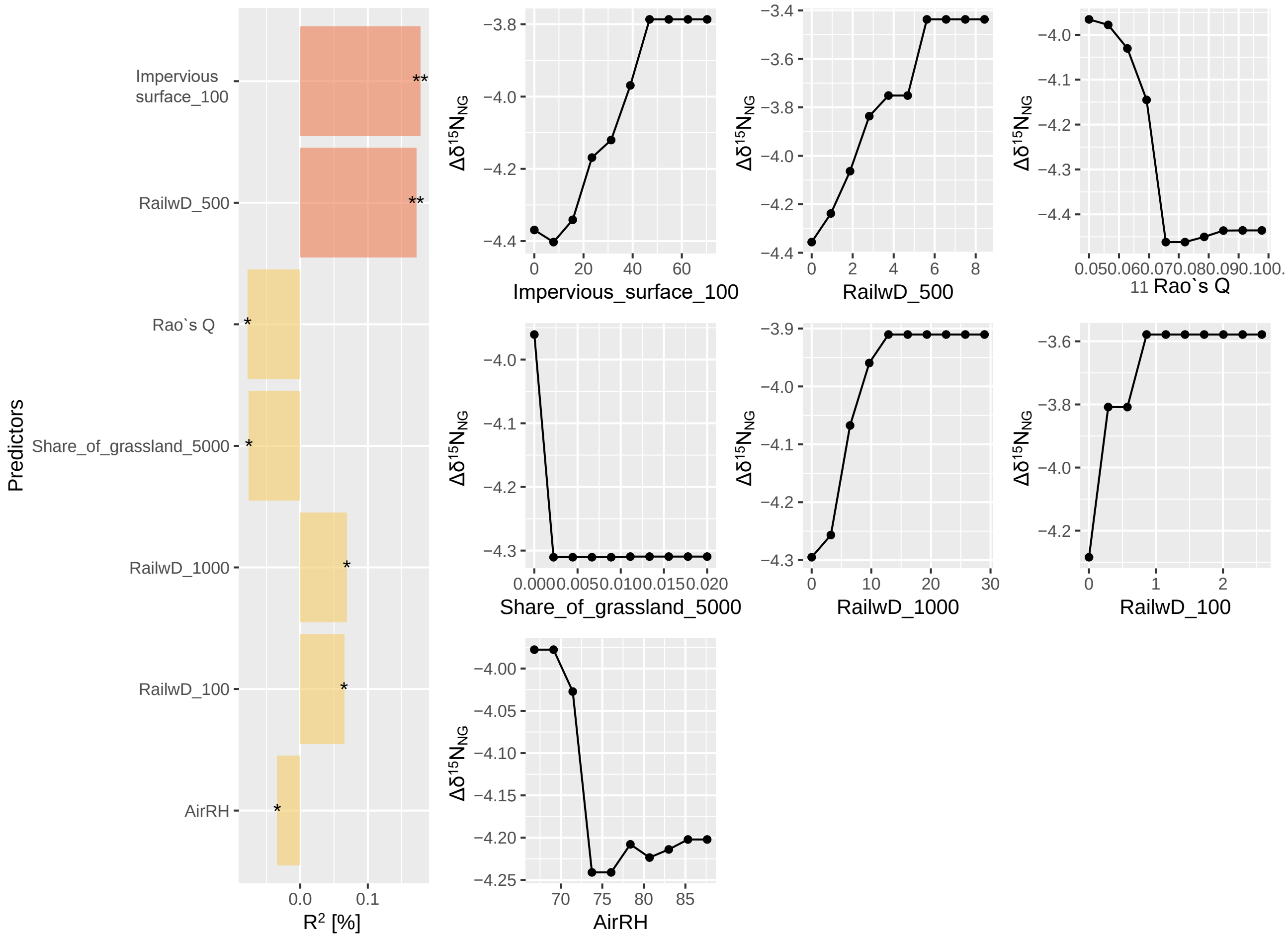
Predictors
Impervious surface_100
RailwD_500
Rao`s Q
Share_of_grassland_5000
RailwD_1000
RailwD_100
AirRH
R² [%]
Δδ15NNG
Impervious_surface_100
RailwD_500
Rao`s Q
Share_of_grassland_5000
RailwD_1000
RailwD_100
AirRH

(G)

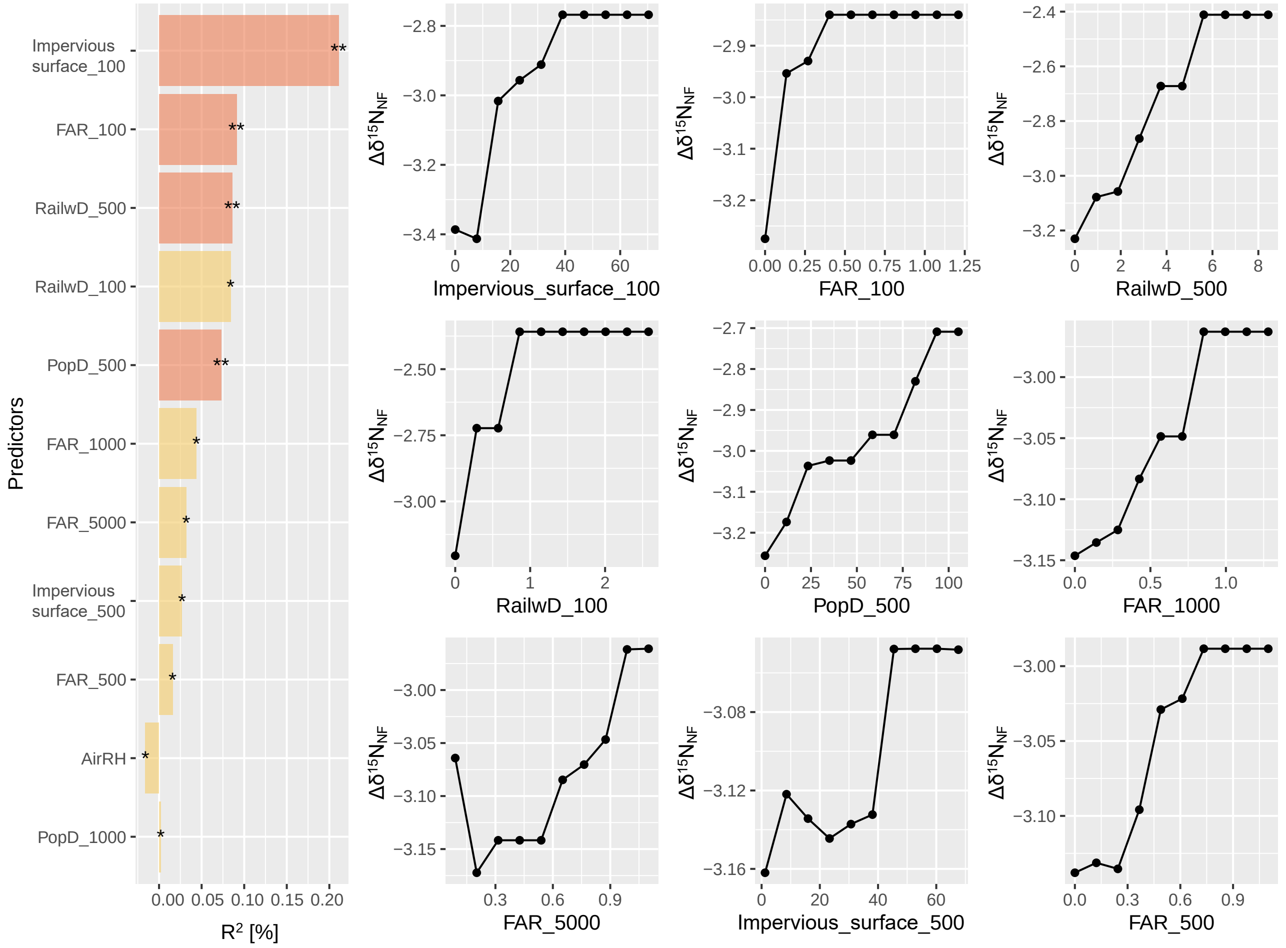
Predictors
Impervious surface_100
FAR_100
RailwD_500
RailwD_100
PopD_500
FAR_1000
FAR_5000
Impervious surface_500
FAR_500
AirRH
PopD_1000
R² [%]
Δδ15NNF
Impervious_surface_100
FAR_100
RailwD_500
RailwD_100
PopD_500
FAR_1000
FAR_5000
Impervious_surface_500
FAR_500

(H)

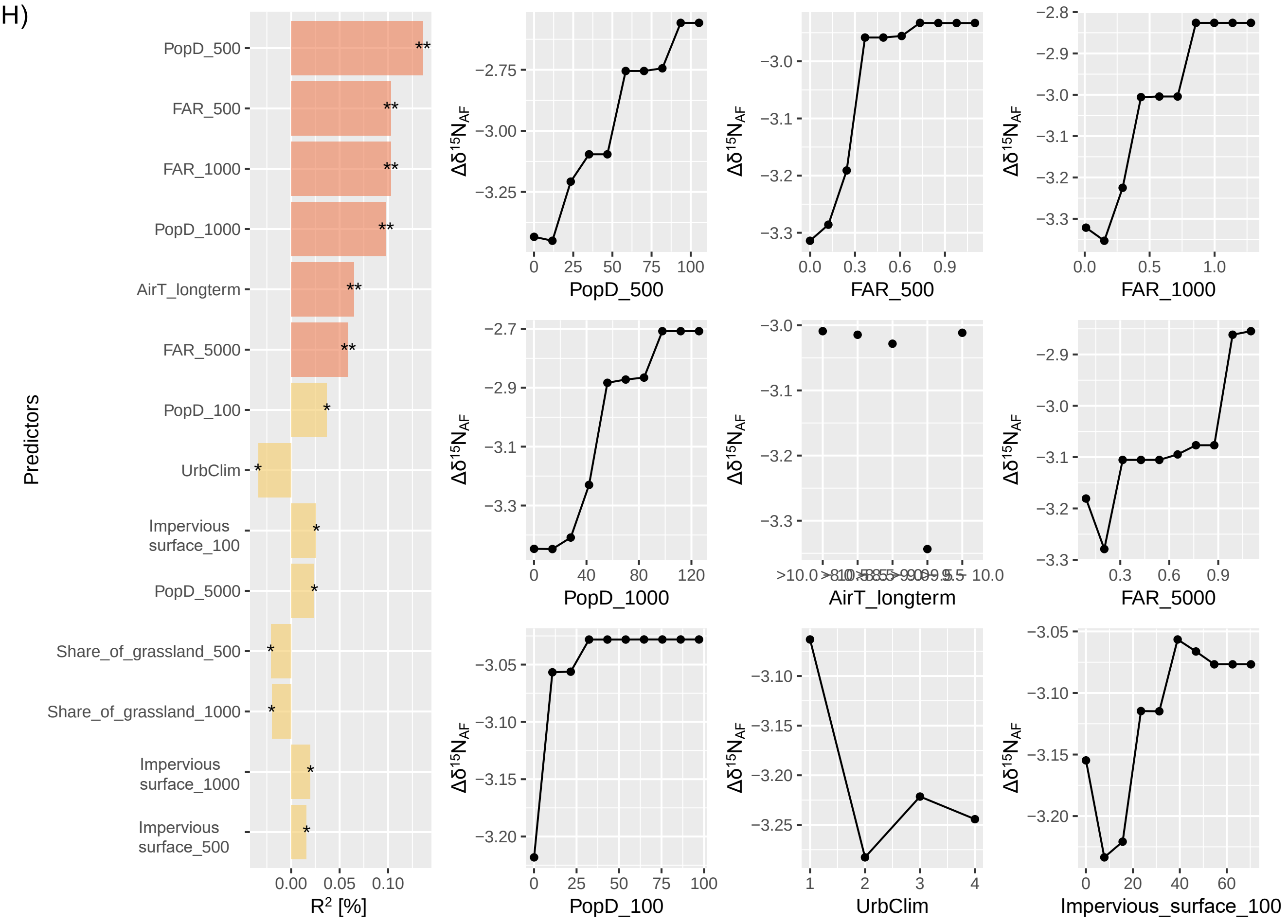
Predictors
PopD_500
FAR_500
FAR_1000
PopD_1000
AirT_longterm
FAR_5000
PopD_100
UrbClim
Impervious surface_100
PopD_5000
Share_of_grassland_500
Share_of_grassland_1000
Impervious surface_1000
Impervious surface_500
R² [%]
Δδ15NAF
PopD_500
FAR_500
FAR_1000
PopD_1000
AirT_longterm
FAR_5000
PopD_100
UrbClim
Impervious_surface_100

S7 File. Partial dependence plots for the predictors selected by random forest models for N enrichment factor ($\Delta\delta^{15}N$) of (A) the whole plant community ($\Delta\delta^{15}N_C$), (B) natives ($\Delta\delta^{15}N_N$), (C) aliens ($\Delta\delta^{15}N_A$), (D) graminoids ($\Delta\delta^{15}N_G$), (E) forbs ($\Delta\delta^{15}N_F$), (F) native graminoids ($\Delta\delta^{15}N_{NG}$), (G) native forbs ($\Delta\delta^{15}N_{NF}$) and (H) alien forbs ($\Delta\delta^{15}N_{AF}$). Note that a partial dependency plot is used not to confirm the effect size but the association pattern including effect direction, since due to normalization, the ranges of y-axes do not directly correspond to the range of the variable. The left panels indicate the relative importance of the explanatory variables selected for each random forest model. The significance of each predictor selected for the models is indicated by asterisks: * $p<0.05$, ** $p<0.01$ and *** $p<0.001$).
